# ﻿Phylogenetic relationships in *Coryphantha* and implications on *Pelecyphora* and *Escobaria* (Cacteae, Cactoideae, Cactaceae)

**DOI:** 10.3897/phytokeys.188.75739

**Published:** 2022-01-21

**Authors:** Daniel Sánchez, Balbina Vázquez-Benítez, Monserrat Vázquez-Sánchez, David Aquino, Salvador Arias

**Affiliations:** 1 CONACYT-Laboratorio Nacional de Identificación y Caracterización Vegetal, Departamento de Botánica y Zoología, Centro Universitario de Ciencias Biológicas y Agropecuarias, Universidad de Guadalajara, Zapopan, Jalisco, C.P. 45220, Mexico; 2 Herbario Luz María Villarreal de Puga, Departamento de Botánica y Zoología, Centro Universitario de Ciencias Biológicas y Agropecuarias, Universidad de Guadalajara, Zapopan, Jalisco, C.P. 45220, Mexico; 3 Programa de Posgrado en Botánica, Colegio de Postgraduados. Carretera México-Texcoco Km 36.5, Montecillo, Texcoco, Estado de México, 56230, Mexico; 4 Jardín Botánico, Instituto de Biología, Universidad Nacional Autónoma de México, Coyoacán, 04510, CDMX, Mexico; 5 Colección de Plantas Suculentas, Facultad de Estudios Superiores Zaragoza, Campus II, Universidad Nacional Autónoma de México, C.P. 15000, CDMX, Mexico

**Keywords:** *Coryphanthamacromeris*, extrafloral glands, groove on tubercule, infrageneric classification, taxonomy

## Abstract

The genus *Coryphantha* includes plants with globose to cylindrical stems bearing furrowed tubercles, flowers arising at the apex, and seeds with flattened testa cells. *Coryphantha* is the second richest genus in the tribe Cacteae. Nevertheless, the genus lacks a phylogenetic framework. The limits of *Coryphantha* with its sister genus *Escobaria* and the infrageneric classification of *Coryphantha* have not been evaluated in a phylogenetic study. In this study we analyzed five chloroplast regions (*matK*, *rbcL*, *psbA-trnH*, *rpl16*, and *trnL-F*) using Bayesian phylogenetic analysis. We included 44 species of *Coryphantha* and 43 additional species of the tribe Cacteae. Our results support the monophyly of *Coryphantha* by excluding *C.macromeris*. *Escobaria* + *Pelecyphora* + *C.macromeris* are corroborated as the sister group of *Coryphantha*. Within *Coryphantha* our phylogenetic analyses recovered two main clades containing seven subclades, and we propose to recognize those as two subgenera and seven sections, respectively. Also, a new delimitation of *Pelecyphora* including *C.macromeris* and all species previously included in *Escobaria* is proposed. To accommodate this new delimitation 25 new combinations are proposed. The seven subclades recovered within *Coryphantha* are morphologically and geographically congruent, and partially agree with the traditional classification of this genus.

## ﻿Introduction

*Coryphantha* (Engelm.) Lem. was described by Engelmann (1856) as a subgenus of *Mammillaria* Haw. Later, Lemaire (1868) raised it to generic level. Hunt and Benson (1976) proposed *Coryphanthasulcata* (Engelm.) Britton & Rose as the type of this genus. *Coryphantha* is morphologically characterized by adult plants with globose to cylindrical stems, covered with numerous spirally-arranged tubercles, flowers that arise at the apex of the stem, stem tubercles with a groove in maturity, and seeds with flattened testa cells (Anderson 2001; Dicht and Lüthy 2005; Hunt et al. 2006). Species of *Coryphantha* mainly inhabit the Mexican highlands in xerophytic shrublands and grasslands, although some prefer tropical deciduous forests and coniferous forests (Dicht and Lüthy 2005).

The taxonomy of *Coryphantha* has been complicated. Attributes such as the shape and size of the stem, the number, color, and orientation of the spines change according to the development state of the specimen, causing confusion with members of other genera such as *Escobaria* Britton & Rose, *Mammillaria*, and *Neolloydia* Britton & Rose (Vázquez-Benítez et al. 2016). For instance, Benson (1969, 1982) recognized *Escobaria* as a subgroup of *Coryphantha* because of the tubercle grooves, an opinion that persists to this day (Zimmerman and Parfitt 2004).

Species number in *Coryphantha* (excluding *Escobaria*) has varied over time, Lemaire (1868) recognized 25 species, Bravo-Hollis and Sánchez-Mejorada (1991) 59 species, Dicht and Lüthy (2001) and Hunt et al. (2006) 43 species, and Vázquez-Benítez et al. (2016) 45 species. This last study was based on a broad and inclusive morphometric analysis (Vázquez-Benítez et al. 2016). Regardless of the differences in species number, *Coryphantha* is the second richest genus in the tribe Cacteae (Vázquez-Benítez et al. 2016).

Current infrageneric classifications in *Coryphantha* have been entirely based on morphology, which has been evaluated according to different criteria, generating artificial classifications. Bravo-Hollis and Sánchez-Mejorada (1991) recognized three series within the genus: *Macromeres* Britton & Rose, *Aulacothelae* Lem., and *Glanduliferae* Salm-Dyck. Dicht and Lüthy (2001, 2005) recognized two subgenera: *Coryphantha* and *Neocoryphantha* Backeb., divided into sections and series. Finally, Hunt et al. (2006) proposed an artificial classification in which three subgenera and three informal groups were recognized. Those proposals have been based on the presence/absence of extrafloral glands at the areole, the type of development and position of the areole on the tubercles, growth form and shape of the tubercle. None of these proposals has been evaluated within a phylogenetic framework.

A previous molecular phylogenetic study of the tribe Cacteae included a few species of the genus *Coryphanta* (Butterworth et al. 2002). This study suggested that *Coryphantha* is part of the *Mammillaria* (=mammilloid) clade, a group that includes other genera such as *Escobaria*, *Neolloydia*, *Ortegocactus* Alexander, and *Pelecyphora* Ehrenb. The position of *Coryphantha* within mammilloid clade was further supported by other studies with better sampling and larger molecular data set (Butterworth and Wallace 2004; Crozier 2005; Bárcenas et al. 2011; Hernández-Hernández et al. 2011; Vázquez-Sánchez et al. 2013). Overall, these phylogenetic studies suggest that *Coryphantha* is not monophyletic (Bárcenas et al. 2011; Vázquez-Sánchez et al. 2013). Recently, Breslin et al. (2021) proposed the recircumscription of the mammilloid clade by recognizing three genera, *Mammillaria*, *Cochemiea* (K.Brandegee), and *Coryphantha* (including *Escobaria*). However, sampling in the *Coryphantha* clade was poor. In this study, we performed phylogenetic analyses focusing on the tribe Cacteae to test for the monophyly of *Coryphantha* and to better understand its relationship to *Escobaria*. With the phylogenetic hypothesis obtained we evaluated the infrageneric classification proposed by Dicht and Lüthy (2005), and propose the set of morphological characters that define the genus *Coryphantha*.

## ﻿Materials and methods

The monophyly of the tribe Cacteae has been largely corroborated by phylogenetic studies (Butterworth et al. 2002; Vázquez-Sánchez et al. 2013). The most comprehensive phylogenetic hypothesis of the tribe recovers three grades and the clade named “core Cacteae”, which is in turn composed by two subclades, the “Ferocactus clade” and the clade B (henceforth “mammilloid clade”) (Vázquez-Sánchez et al. 2013). The present comprehensive study included 44 species of *Coryphantha* (95.6%), eight species of *Escobaria* (44%), 30 additional taxa belonging to the “mammilloid clade”, four taxa of the “Ferocactus clade”, 10 taxa of the “Sclerocactus clade”, and *Echinocactusplatyacanthus* Link & Otto as the functional outgroup (Appendix 1). For the genus *Coryphantha*, we followed the species accepted by Dicht and Lüthy (2005) and those accepted by Arias et al. (2012). Our analyses included mostly new sequences for *Coryphantha* and complementary sequences previously published (Butterworth et al. 2002; Butterworth and Wallace 2004; Bárcenas et al. 2011; Hernández-Hernández et al. 2011; Fehlberg et al. 2013; Schwabe et al. 2015; Kuzmina et al. 2017; Aquino et al. 2019, and Vázquez-Sánchez et al. 2013, 2019) (Appendix 1).

Samples of plant tissue from the epidermis and hypodermis of the stem were dried, frozen, and pulverized. Total DNA extraction was achieved by using the DNeasy plant mini kit (Qiagen, California). We amplified chloroplast markers widely used in phylogenetic reconstruction in Cacteae (Vázquez-Sánchez et al. 2013, 2019). Specifically, we amplified the chloroplast coding regions *mat*K and *rbc*L, and the intergenic spacers *psb*A-*trn*H and the *trnL-trn*F (including part of the *trn*L), and the *rpl*16 intron. Primers and profiles of thermal cycles followed Vázquez-Sánchez et al. (2013). The PCR products were sequenced at the High Throughput Genomics Unit at the University of Washington (now unavailable). Appendix 1 shows in detail the GenBank accessions for each taxon.

The sequences for each marker were assembled using SEQUENCHER (v. 4.8, Gene Codes Corporation 2007). The matrices were aligned manually with MESQUITE (v. 2.75, Maddison and Maddison 2015). Table 1 shows some numeric records about the taxa and the aligned sequences included in the subsequent analyses. Insertion-deletion events in aligned sequences (indels) were coded using the simple coding method (Simmons and Ochoterena 2000) (Appendix 2). Additionally, eight morphological characters, proposed as diagnostic for *Coryphantha* and related genera were coded and used in a combined phylogenetic analysis. It has been suggested that in Cactaceae the inclusion of indels and a set of morphological characters in phylogenetics analysis results in more accurate hypotheses (Sánchez et al. 2019; Martínez-Quezada et al. 2020). Character states were extracted from the descriptions of the species (Bravo Hollis and Sánchez-Mejorada 1991; Barthlott and Hunt 2000; Dicht and Lüthy 2005; Hunt et al. 2006) and corroborated in the field, in living collections (Jardín Botánico, Instituto de Biología, UNAM), and with herbarium specimens (MEXU). Characters and character states are listed in Table 2. DNA evolution models for each sequence were estimated using the corrected Akaike information criterion (AICc) in JMODELTEST2 (Darriba et al. 2012) on the CIPRES Science Gateway (v. 3.3 Miller et al. 2010) (Table 1). The Mkv model (Lewis 2001) was assigned for the indels and the morphological partitions. The first matrix was concatenated by including the five DNA regions. The second matrix included the five DNA regions and the indels partition. Finally, the third matrix included the five DNA regions, the indels and morphological characters. A partitioned Bayesian inference (BI) analysis was performed in MRBAYES (v. 3.2.1, Ronquist et al. 2012). The BI analysis for those matrices consisted of two runs of four chains for 20 million iterations, saving one tree every 1000 generations, and beginning with one random tree. The burn-in parameter was fixed as 25%.

**Table 1.** Data of the aligned sequences used in the phylogenetic analysis.

	*matK*	*psbA-trnH*	*rcbL*	*rpl16*	*trnL-F*	Combo
Taxa	95/99	91/99	83/99	86/99	85/99	–
Length (aligned)	817	391	538	1349	1218	4313
Non-informative sites	730	313	509	1100	1048	3700
Informative sites	87	78	29	249	170	613
% informative sites	10.6	19.9	5.4	18.4	13.9	14.2
Informative indels	1	11	0	8	14	34
Substitution model	TPM1uf+I+G	TPM1uf+I+G	K80+I	TIM1+I+G	TVM+G	–

The ancestral states of the eight morphological characters were traced in the selected phylogeny to test them as potential synapormophies of clades. The tracing of characters was performed in MESQUITE (v.2.75, Maddison and Maddison 2015) using the parsimony ancestral state method on the majority consensus tree from the combined BI analysis.

**Table 2.** Characters and character states for the ancestral states reconstruction.

1. Growth form: (0) globose, (1) short cylindrical, (2) cylindrical, (3) depressed-globose.
2. Groove on tubercle in mature plant: (0) absence, (1) complete, (2) incomplete.
3. Extrafloral glands at or near the axil: (0) absence, (1) turgid throughout the year, (2) turgid only at flowering season.
4. Position of the flowers: (0) apical or nearly apical, (1) in a ring distant from the apex.
5. Margin of the outer tepals: (0) fimbriate, (1) entire.
6. Color of the mature fruit: (0) red-pink, (1) green, (2) yellow.
7. Type of cortex: (0) watery, (1) mucilaginous, (2) laticiferous.
8. Multicellular sculpture of the lateral side of the seed: (0) flat, (1) tuberculate, (2) pitted.

## ﻿Results

**Figure 1.**
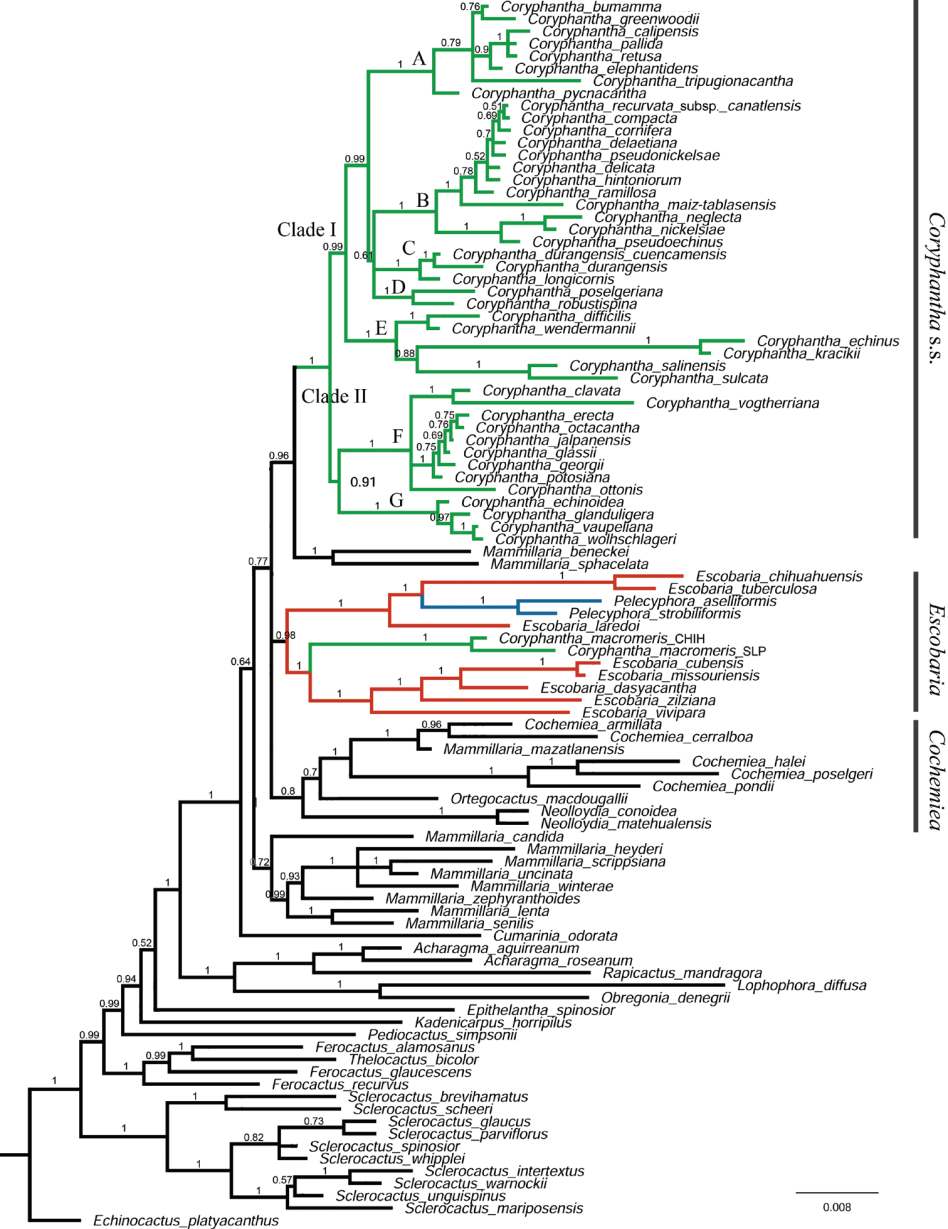
Phylogenetic relationships of *Coryphantha* and close related genera. Majority rule phylogram, from the BI analysis using cpDNA sequences and indels partitions (molecular analysis). Numbers in nodes indicate posterior probabilities. Labels indicate the main recovered clades and subclades.

**Figure 2.**
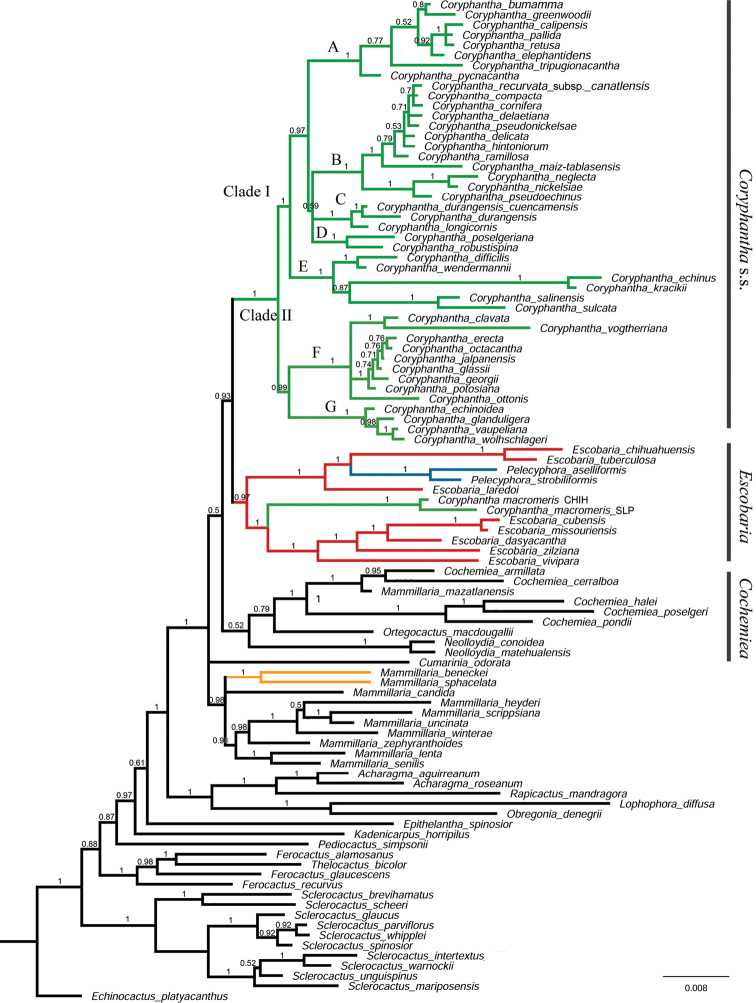
Phylogenetic relationships of *Coryphantha* and close related genera. Majority rule phylogram, from the BI analysis using cpDNA sequences, indels, and morphological partitions (combined analysis). Numbers in nodes indicate posterior probabilities. Labels indicate the main recovered clades and subclades.

Phylogenetic analyses including DNA sequences only (Appendix 3: Fig. A1) and DNA sequences + indels partition (henceforth “molecular analysis”) showed identical topologies (Fig. 1). The phylogenetic analysis with morphological data (henceforth “combined analysis”) recovered a more resolved phylogeny (Fig. 2) with minor changes in the main clades, except for the position of one clade. In the molecular analysis, *Mammillariasphacelata* and *M.beneckei* were recovered as the sister clade to *Coryphantha* s.s. (PP = 0.96, Fig. 1). This clade formed a polytomy with *Cochemiea* and *Escobaria* (including *Pelecyphora*) clades (Fig. 1). In the combined analysis, *M.sphacelata* and *M.beneckei* were included in the *Mammillaria* clade PP = 0.98, Fig. 2). Each clade; *Cumarinia*, *Mammillaria*, *Cochemiea*, *Escobaria*, and *Coryphantha* s.s. showed resolved relationships between them with moderate to low support (Fig. 2).

In all analyses the *Cochemiea* clade included *Mammillariamazatlanensis* (PP = 1.0) *Ortegocactusmacdougallii* (PP = 0.7, Fig. 1; PP = 0.79, Fig. 2), and *Neolloydia* (PP = 0.8, Fig. 1; PP = 0.52, Fig. 2). Phylogenetic relationships in both analyses indicate that *Coryphantha* is not a monophyletic group, because *C.macromeris* was recovered in the *Escobaria* clade (Figs 1, 2). *Coryphantha* s.s. is divided into two main clades, with 33 species grouped in clade I (PP = 0.99, Fig. 1; P = 1, Fig. 2), and 13 species grouped in clade II (PP = 0.91; Fig. 1; PP = 0.99. Fig. 2). Clade I is composed by five subclades (A, B, C, D, E), and Clade II by two subclades (F, G) (Figs 1, 2), all of them with high supports. The *Escobaria* clade (PP = 0.98, Fig. 1; PP = 0.97, Fig. 2) is divided into two subclades, the first one includes *Coryphanthamacromeris*, *Escobariacubensis*, *E.dasyacantha*, *E.missourensis*, *E.vivipara*, and *E.zilziana* (PP = 1, Figs 1, 2); while the second subclade includes *E.laredoi*, *Pelecyphoraasselliformis*, *P.strobiliformis*, *E.tuberculosa*, and *E.chihuahuensis* (PP = 1.0; Figs 1, 2).

The ancestral state reconstruction (Appendix 3: Fig. A1) showed that the presence of a complete groove on the tubercle (Appendix 3: Fig. A1B), the apical origin of the flowers (Appendix 3: Fig. A1D), the entire margin of the outer tepals (Appendix 3: Fig. A1E), the green color of the fruit (Appendix 3: Fig. A1F), and the flat multicellular sculpture of the lateral side of the seed (Appendix 3: Fig. A1H) were ancestral states to *Coryphantha* s.s., few or null changes on these characters states occurred inside the clade. In contrast, in the *Escobaria* clade, the fimbriate margin of the outer tepals (Appendix 3: Fig. A1E), the red color of the mature fruit (Appendix 3: Fig. A1F), and the pitted multicellular sculpture of the seed were ancestral character states (Appendix 3: Fig. A1H). Additionally, growth form was ambiguous in *Coryphantha* s.s. and *Escobaria* clade. The absence of glands near the axil of the tubercles was ancestral to *Coryphantha* s.s., and the presence of those glands evolved independently in two subclades of *Coryphantha* (Appendix 3: Fig. A1C). In clade II, turgid glands present all year-long were ancestral, while glands present only during flowering season evolved once in subclade D (Appendix 3: Fig. A1C. Finally, watery cortex was ancestral in *Corypantha* s.s., but it changed into mucilaginous cortex in the subclade F (Appendix 3: Fig. A1G).

## ﻿Discussion

The close relationships among *Cochemiea*, *Coryphanta*, *Cumarinia*, *Escobaria*, and *Mammillaria* have been recognized by several studies (Butterworth and Wallace 2004; Crozier 2005; Vázquez-Sánchez et al. 2013; Breslin et al. 2021). Breslin et al. (2021) recovered them as closely related lineages and redefined their limits. These authors proposed to expand the limits of *Cochemiea* to include 37 species of *Mammillaria*, *Neolloydia*, and *Ortegocactus*. Our results (Figs 1, 2) recovered, with moderate to low support, the same phylogenetic position of *Ortegocactus* and *Neolloydia*. Additionally, *Mammillariamazatlanensis* was nested within *Cochemiea*. Morphological (Hunt 1985) and molecular evidence (Butterworth and Wallace 2004) suggest that *M.mazatlanensis* is closely related to other taxa now classified within *Cochemiea*, so it should be transferred (see Taxonomic summary).

In the molecular analysis, *Mammillariasphacelata* and *M.benecki* were recovered, with low support, as the sister group to *Coryphantha* s.s. In contrast, Breslin et al. (2021) found *M.sphacelata* to be the sister to *Escobaria* + *Coryphantha*. The addition of eight morphological characters in the combined analysis recovered *M.sphacelata* and *M.beneckei* within the clade *Mammillaria*, and supported *Coryphantha* s.s. and *Escobaria* as sister lineages. We argue that the low sampling of this early diverged lineage of *Mammillaria* (Butterworth and Wallace 2004) and the few sequences included do not allow us to conclude about their relationships.

Finally, Breslin et al. (2021) proposed *Escobaria* and *Coryphantha* to be a single genus, as traditionally treated by North American botanists (Benson 1982; Zimmerman and Parfitt 2004). However, sampling in Mexican *Coryphantha* was not representative. Molecular and combined analyses recovered *Coryphantha* and *Escobaria* as independent lineages and the ancestral state reconstruction (Appendix 3: Fig. A1) showed that each genus has a unique combination of morphological characters. Our results support the traditional recognition of *Coryphantha* and *Escobaria* as separate genera (Taylor 1979; Bravo-Hollis and Sánchez Mejorada 1991; Dicht and Lüthy 2005; Hunt et al. 2006; Korotkova et al. 2021).

### ﻿*Escobaria* clade

**Figure 3.**
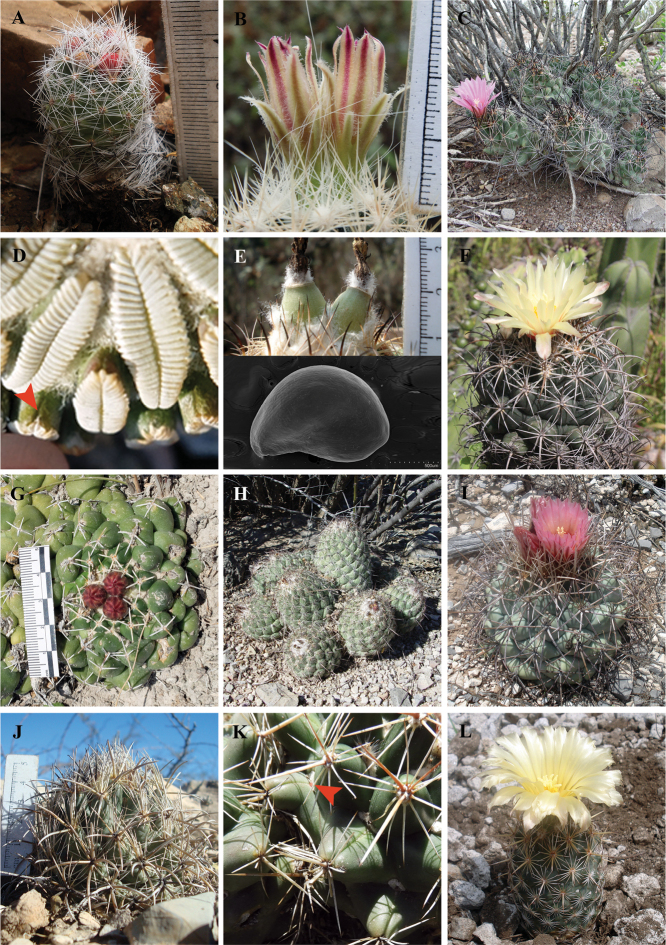
Representative species and morphology of *Coryphantha* and *Escobaria***A***Escobariadasyacantha* bearing red fruits (*S. Arias 2090*, MEXU) **B** flower of *Escobariaemskoetteriana* (Quehl) Borg with fimbriate outer tepals (*D. Aquino 322*, MEXU) **C***Coryphanthamacromeris* bearing flowers with fimbriate outer tepals (*S. Arias 1788*, MEXU) **D** close-up of the furrow on the tubercles (arrow) in *Pelecyphoraaselliformis* (*H. Sánchez-Mejorada 3616*, MEXU) **E** green fruits (top) and flat multicellular sculpture of the lateral side of the seed (bottom) in *Coryphanthacalipensis* (*B. Vázquez 2555*, MEXU) **F***Coryphanthamaiz-tablasensis* (*D. Aquino 400*, MEXU) **G***Coryphanthacornifera* (*SA 2212*, MEXU) **H***Coryphanthadurangensis* (*B. Vázquez 2625*, MEXU) **I***Coryphanthaposelgeriana* (*S. Arias 2109*, MEXU) **J***Coryphanthakracikii* (*B. Vázquez* 2618, MEXU) **K** glands at the axil (arrow) in *Coryphanthaottonis* (*D. Sánchez* s.n., IBUG) **L***Coryphanthaglanduligera* (*S. Arias 2129*, MEXU).

The eight sampled species of *Escobaria*, together with *Coryphanthamacromeris*, *Pelecyphoraaselliformis*, and *P.strobiliformis* form a monophyletic group with high support values (Figs 1, 2). This clade is diagnosed by the tubercles with complete grooves, external tepals with fimbriate margins, and seeds with pitted multicellular sculpture on the lateral side (except in *C.macromeris*, and *Escobariachihuahuensis*) (Appendix 3: Fig. A1, Fig. 3).

Although previous molecular analyses recovered *C.macromeris* outside the core *Coryphantha* clade, phylogenetic relationships of *C.macromeris* were not clear due to lack of resolution (Bárcenas et al. 2011) and insufficient sampling of *Coryphantha* (Vázquez-Sánchez et al. 2013; Crozier 2005). Our analyses, including 46 taxa of *Coryphantha*, recovered two different samples of *C.macromeris* in the *Escobaria* clade (PP = 1.0, Figs 1, 2), contrasting with the traditional classification in the monotypic sectionLepidocoryphantha (Backeberg) Moran, or subgenusNeocoryphantha Backeb. (sensu Dicht and Lüthy 2005). Previous morphological analysis of *Coryphantha* concluded that *C.macromeris* was the most dissimilar taxon of the genus *Coryphantha* (Vázquez-Benítez et al. 2016). The main character that differentiates this species from the rest of the species in the *Coryphantha* clade is the presence of an incomplete groove in the tubercles and fimbriate outer tepals.

*Coryphanthamacromeris* shares the fimbriate outer tepals with the other species of the genus *Escobaria* (Fig. 3B, C). Interestingly, *C.macromeris* and *Escobariavivipara* show identical flower morphology (Zimmerman and Parfitt 2004). Additionally, *E.chihuahuensis* shows a shallowly pitted lateral seed coat (Barthlott and Hunt 2000, plate 73.3–4), similar to the flat cells observed in *Coryphantha*. Probably, the flat sculpture of the lateral side of the seed in *C.macromeris* is the result of the same development observed in *E.chihuahuensis*. As observed in *Ferocactus* (Taylor and Clark 1983) the change of pitted to flat relief of periclinal walls of the seed testa has evolved independently in several lineages of the tribe Cacteae (Appendix 3: Fig. A1H). Given our results, we propose the recognition of *C.macromeris* as a member within the new rearrangement of *Escobaria* and *Pelecyphora* described in the following paragraphs (see Taxonomic summary).

As in previous analysis our phylogenetic hypothesis recovered the two species of *Pelecyphora* in the *Escobaria* clade (Butterworth and Wallace 2004; Bárcenas et al. 2011; Vázquez-Sánchez et al. 2013). Traditionally, *Pelecyphora* is recognized (Boke 1959; Anderson and Boke 1969) by the presence of a rudimentary groove on the tubercles and the “reticulate or striate” seed structure (“par-concave” sensu Barthlott and Hunt 2000). However, *Pelecyphora* also falls into Taylor’s (1979) concept of *Escobaria*, which is circumscribed by seeds with intracellular pits (par-concave) and grooved tubercles. Following Boke (1959), the rudimentary groove in *Pelecyphora* (Fig. 3D) is morphologically equivalent to the groove found on the tubercles of *Coryphantha* and *Escobaria*. Regarding seed morphology, the pitted appearance of the seed coat in *Escobaria* happens because only the central portion of the outer wall of the testa cell is thinner and collapses, while in *Pelecyphora* the entire outer wall of the testa cell is thin and collapses (Barthlott and Hunt 2000). Therefore, *Escobaria* and *Pelecyphora* show a pitted lateral seed coat, differing in cell shape and pit diameter.

Finally, the margin of the outer tepals in *P.aselliformis* may be entire or fimbriate, while in *P.strobiliformis* is always fimbriate (Anderson and Boke 1969); this character is also observed in all species of *Escobaria* (Zimmerman and Parfitt 2004; Hunt et al. 2006). We hypothesized that *Pelecyphora* represents a derived lineage in *Escobaria* that has changed radically its growth form and the shape of its tubercules to occupy specific niches in the Sierra Madre Oriental. A similar trend is observed in species of the genus *Turbinicarpus* (Backeb.) Buxb. & Backeb., in which some species have evolved into a globose-depressed growth form with cylindrical and flattened distally (hatchet-shaped) tubercles (e.g., *Turbinicarpuspseudopectinatus* (Backeb.) Glass & R.A.Foster) or pyramidal and dorsiventrally flattened (scale-like) tubercles (e.g., *Turbinicarpusschmiedickeanus* (Boed.) Buxb. & Backeb.) (Vázquez-Sánchez et al. 2019).

Several studies recovered with high support the alliance of *Pelecyphora* and a clade including *Escobariatuberculosa*, the type species of *Escobaria*. A diagnostic character of *Escobaria* and *Pelecyphora* is the outerperianth-segments with ciliated margins as shown in *E.emskoetteriana* (Fig. 3B), *E.abdita* Řepka & Vaško (Řepka and Vaško 2011) and *E.sneedi* Britton & Rose (Benson 1982) not included in this analysis. The genus *Pelecyphora* was published in 1843 by Ehrenberg, while *Escobaria* was published 80 years later, in 1923, by Britton and Rose. In this context, we propose to merge *Escobaria* members, including *C.macromeris* into *Pelecyphora* (see Taxonomic summary) following priority of publication as dictated by the principle III of the International Code of Nomenclature for algae, fungi, and plants (Turland et al. 2018).

### ﻿*Coryphantha* clade

*Coryphantha* can be recognized as a natural group by excluding *C.macromeris*. *Coryphantha* s.s. (henceforth *Coryphantha*) conformed a robust clade (PP = 1, Figs 1, 2) and can be diagnosed by tubercles with a complete groove, flowers with apical origin, outer tepals with entire margin, green fruits, and seed with flat multicellular sculpture on the lateral side (Appendix 3: Fig. A1, Fig. 3).

Although subgenera *Neocoryphantha* and *Coryphantha* recognized by Dicht and Lüthy (2005) are partially recovered, our phylogenetic analyses showed that most of the infrageneric sections and series proposed by these authors do not represent natural entities. All sampled members of subgenusCoryphantha were recovered in clade I, including taxa without turgid glands near the axil throughout the year (Appendix 3: Fig. A1C). However, this clade also included two of the species assigned to section Robustispina Dicht & A. Lüthy in the subgenusNeocoryphantha (Table 3), making CoryphanthasubgenusCoryphantha (sensu Dicht and Lüthy 2005) a paraphyletic group. Clade II grouped all the members of the subgenus NeocoryphanthasectionNeocoryphantha, but the members of the sections *Lepidocoryphantha* and *Robustispina* (Fig. 1) were recovered in the clade *Escobaria* and the clade I, respectively. Therefore, CoryphanthasubgenusNeocoryphantha (sensu Dicht and Lüthy 2005) represents a polyphyletic group. All members of clade II show turgid glands at or near the axil throughout the year (Fig. 3K), which is recognized as a consistent diagnostic feature and a potential synapomorphy for this lineage (Appendix 3: Fig. A1C).

**Table 3.** Species memberships of the main clades obtained in this study and their previous infrageneric classification by Dicht and Lüthy (2005).

Clade I	subgenusCoryphantha and subgenus NeocoryphanthasectionRobustispina
Subclade A	Series *Retusae*: *Coryphanthaelephantidens* complex and *C.retusa.*
Subclade A	Series *Pycnacanthae*: *C.pycnacantha* and *C.tripugionacantha*
Subclade A	Series *Salinenses* (in part): *C.pallida* complex
Subclade B	Series *Coryphantha* (in part): *Coryphanthahintoniorum* and *C.maiz-tablasensis*
Subclade B	Series *Corniferae* (in part): *C.compacta*, *C.cornifera*, *C.delaetiana*, *C.delicata*, *C.echinusC.neglecta*, *C.nickelsiae*, *C.pseudoechinus*, *C.pseudonickelsiae*, *C.ramillosa*, and C.recurvatasubsp.canatlanensis
Subclade C	Series *Salinenses* (in part): *Coryphanthadurangensis*, C.durangensissubsp.cuencamensis, and *C.longicornis*
Subclade D	sectionRobustispina: *Coryphanthaposelgeriana* and *C.robustispina*
Subclade E	Series *Coryphanta* (in part): *C.sulcate*
Subclade E	Series *Salinenses* (in part): *C.difficilis*, *C.kracikii*, and *C.salinensis*
Subclade E	Series *Corniferae* (in part): *C.werdermannii* and *C.echinus*
Clade II	Subgenus Neocoryphantha except sectionRobustispina
Subclade F	Series *Clavatae*: *C.octacantha*, *C.jalpanensis*, *C.clavata*, *C.clavata*, *C.glassii*, *C.erecta*, and *C.potosiana*
Subclade F	Series *Ottonis*: *C.ottonis*, *C.vogtherriana*, and *C.georgii*
Subclade G	Series *Echinoideae*: *C.wohlschlageri*, *C.vaupeliana*, *C.glanduligera*, and *C.echinoidea*

In order to reflect the relationships found in our phylogenetic hypothesis and to provide a natural infrageneric classification of the genus, we re-circumscribe the two subgenera in *Coryphantha*. One for clade I, the subgenusCoryphantha, and another one for clade II, the subgenusNeocoryphantha (see Taxonomic summary). We further propose to recognize the recovered subclades as sections (see Taxonomic summary). The species belonging to each section, their morphological similarities, and their distribution (biogeographic provinces) are discussed below.

CoryphanthasubgenusCoryphantha (clade I) emerged in five subclades that partially represent some taxonomic groups proposed by Dicht and Lüthy (2005). However, series and subseries suggested by these authors do not represent monophyletic groups. Clade A included species from series *Retusae* Dicht & A. Lüthy, *Pycnacanthae* Dicht & A. Lüthy and *Salinenses* Dicht & Lüthy (Table 3). In this case, members of clade A present most of the radial spines with subulate shape (Fig. 3F) (Bravo-Hollis and Sánchez-Mejorada 1991; Dicht and Lüthy 2005). Our results found that the species complexes *C.elephanthidens* and *C.pallida* do not represent monophyletic groups. This result corroborates that *C.bumamma* and *C.greenwoodii* are different species from *C.elephantidens* as proposed by Vázquez-Benítez et al. (2016). Additionally, our results support the proposal of Arias et al. (2012) to recognize *C.calipensis* and *C.pallida* as two distinct species. The distinction of *C.pseudoradians* Bravo from *C.pallida* Britton & Rose, remains unresolved, since the former was not included in our analysis.

As documented by Dicht and Lüthy (2005), there was a historical confusion between *C.pycnacantha* and *C.pallida*, since they are morphologically similar (Arias et al. 2012). This affinity is now justified since they belong to the same clade. Dicht and Lüthy (2005) classified *C.pallida* within series *Salinensis* along with northern species. This species emerged in Clade A, which is recognized here as sectionRetusae (see Taxonomic summary). This is distributed in central Mexico, encompassing the southern portion of the piedmont of Sierra Madre Occidental, the Mexican High Plateau, the plains and piedmonts of the Mexican Transvolcanic Belt, the southern portion of Sierra Madre Oriental, and the Balsas Basin.

Clade B included members of the series *Coryphantha* and *Corniferae* Dicht & A. Lüthy (Table 3). Members of this clade show upright or radiate tubercles (Fig. 3G). This lineage is recognized in the present work as the sectionCorniferae. This clade presents a wide distribution and occupies several northern ecoregions. An eastern group of species inhabits the Chihuahuan Desert, the Sierra Madre Oriental, and the Tamaulipas-Texas Semiarid Plain, and a western group occupies the Chihuahuan Desert, the piedmont of the Sierra Madre Occidental, and the Sierra Madre Occidental.

*Coryphanthagracilis* is classified into the monotypic sectionGracilicoryphantha Dicht & Lüthy by the presence of globose seed and broad basal hylum (Dicht and Lüthy 2005). Although *C.gracilis* was not included in our analysis, we suggest that it belongs to clade B, because of its morphological affinity to *C.compacta* and *C.recurvata* (Vázquez-Benítez et al. 2016), and also the similar geographic distribution. *Coryphanthapulleineana* (Backeb.) Glass was not included in our analysis. Dicht and Lüthy (2005) mention some morphological affinities to *C.ramillosa*. In addition, *C.pulleineana* and *C.pseudoechinus* shared the presence of glands in the spiniferous areole. For now, we propose *C.pulleineana* as a member of this group because of its morphological and geographical congruence to other species of this clade (Dicht and Lüthy 2005).

Subclade C included two members of the series *Salinenses* (Table 3). These taxa can be distinguished by the presence of appressed tubercles and woolly stem tips (Fig. 3H) (Bravo-Hollis and Sánchez-Mejorada 1991; Dicht and Lüthy 2005). Our study included C.durangensissubsp.durangensis and C.durangensissubsp.cuencamensis, which formed a monophyletic group. However, they showed different branch lengths, which suggests that its recognition as different species, as proposed by Vázquez-Benítez et al. (2016), must be considered. This small group is recognized in the present work as the sectionDurangenses (see Taxonomic summary). This group presents a narrow distribution in the state of Durango, inhabiting the Chihuahuan Desert and the piedmont of the Sierra Madre Occidental.

Subclade D corresponds to CoryphanthasectionRobustispina (Table 3, Taxonomic summary). This clade is supported by the presence of turgid glands near the axil only during the flowering season (Fig. 3I; Appendix 3: Fig. A1C). Although those species have been grouped in the past with other taxa with glands (Dicht and Lüthy 2005; Vázquez-Benítez et al. 2016), our results suggested that this character state emerged independently from an ancestral with absent glands. This species occurs in the Chihuahuan Desert and in the northern piedmont of the Sierra Madre Occidental.

Subclade E was formed by six taxa classified into the series *Coryphanta*, series *Salinenses*, and series *Corniferae* (Table 3). There are no evident morphological characters that define clade C. Affinities such as the red filaments have been observed in *C.echinus*, *C.kracikii*, *C.salinensis*, and *C.sulcata*. Particularly, *C.salinensis* and *C.sulcata* share a yellow flower with a brilliant red flower throat (Dicht and Lüthy 2005). Also, *C.difficilis*, *C.kracikii*, *C.salinensis* show tubercles appressed, and slightly appressed in *C.werdermannii* (Fig. 3J). Members of subclade E are proposed here as the CoryphanthasectionCoryphantha, which is distributed in the Chihuahuan Desert, the Sierra Madre Oriental, and the Tamaulipas-Texas Semiarid Plain.

We propose the division of subgenusNeocoryphantha (clade II) into two sections. The first one is sectionClavatae (see Taxonomic summary), which corresponds to subclade F (Table 3). This section presents mucilaginous cortex (Dicht and Lüthy 2005), a character recovered as ancestral to the group in our analyses (Fig. 3K, Appendix 3: Fig. A1G). SectionClavatae occurs mainly in the southern part of the Chihuahuan Desert and in the Mexican High Plateau, with *C.ottonis* ranging to the interior plains and piedmonts of the Sierra Madre Occidental and the Mexican Transvolcanic Belt. The second is sectionEchinoideae, which corresponds to subclade G (Fig. 3L, Table 3). This section can be recognized by the presence of watery cortex (Appendix 3: Fig. A1G). Members of the section are distributed in the Chihuahuan Desert and the Sierra Madre Oriental.

## ﻿Taxonomic summary

### ﻿*Cochemiea*

Phylogenetic analyses support the addition of *Mammillariamazatlanensis* within *Cochemiea*. Three lectotypes are proposed.

#### *Cochemiea* (K.Brandegee) Walton. Cact. J. (London) 2: 50. 1899.

##### ﻿ Cochemiea mazatlanensis

Taxon classificationPlantaeCaryophyllalesCactaceae

(K.Schum.) D.Aquino & Dan.Sanchez comb. nov.

urn:lsid:ipni.org:names:77248940-1

≡ Mammillariamazatlanensis K.Schum., Monatsschr. Kakteenk. 11: 154. 1901.Neomammillariamazatlanensis (K.Schum.) Britton & Rose, Cactaceae 4: 138. 1923. Chilitamazatlanensis (K.Schum.) Orcutt, Cactography 2. 1926. Ebnerellamazatlanensis (K.Schum.) Buxb., Oesterr. Bot. Z. 98: 89. 1951. Escobariopsismazatlanensis (K.Schum.) Doweld, Sukkulenty 3: 40. 2000. Type: México, Sinaloa, Matzatlán [Mazatlán], *W. Mundt s.n.* (not preserved, lectotype, designated here, Monatsschr. Kakteenk. 15: 154. 1905: Illustration “*Ma*[*m*]millaria mazatlanensis K.Schum. Nach einer von Herrn Mundt für die “Monatsschrift für Kakteenkunde” hergestellten Photographie”). **Notes.** Both the original description of Mammillaria (= Cochemiea) mazatlanensis (Schumann 1901), and the later extension of the description by Gurke (1905) do not indicate that a type specimen has been preserved. Hunt (1985) confirms that a type specimen was not formally designated.
= Mammillarialittoralis K.Brandegee, Bull. Misc. Inform. Kew 1908: App. 91. 1908. Type: Not designed.
= Neomammillariaoccidentalis Britton & Rose, Cactaceae 4: 161–162, f. 179. 1923. Chilitaoccidentalis (Britton & Rose) Orcutt, Cactography 2. 1926. Mammillariaoccidentalis (Britton & Rose) Boed., Mammillarien-Vergleichs-Schluessel: 36. 1933. Ebnerellaoccidentalis (Britton & Rose) Buxb., Oesterr. Bot. Z. 98: 90. 1951. Mammillariamazatlanensisvar.occidentalis (Britton & Rose) Neutel., Succulenta (Netherlands) 65: 119. 1986. Type: México, Colima, near Manzanillo, Dec 1890, *E. Palmer 1053* (holotype: US [208544 image!]).
= Neomammillariasinaloensis Rose, Fl. Indig. Sinaloa Cact.: 3. 1929. Nom. Inval.
= Neomammillariapatonii Bravo, Anales Inst. Biol. Univ. Nac. Mexico 2: 129. 1931. Mammillariapatonii (Bravo) Werderm., Backeberg, Neue Kakteen: 97. 1931. Mammillariaoccidentalisvar.patonii (Bravo) R.T.Craig, Mammill. Handb.: 169. 1945. Mammillariamazatlanensisf.patonii (Bravo) Neutel., Succulenta (Netherlands) 65: 119. 1986. Mammillariamazatlanensissubsp.patonii (Bravo) D.R.Hunt, Mammillaria Postscripts 7: 3. 1998. Escobariopsismazatlanensissubsp.patonii (Bravo) Doweld, Sukkulenty 3: 41. 2000. Type: México, Sinaloa [Nayarit], Isla Tres Marías 1930, Heilfurth s.n. (MEXU).
= Mammillariaoccidentalisvar.sinalensis R.T.Craig, Mammill. Handb.: 169. 1945. Mammillariapatoniivar.sinalensis (R.T.Craig) Backeb., Cactaceae 5: 3291. 1961. Mammillariamazatlanensisf.sinalensis (R.T.Craig) Neutel., Succulenta (Netherlands) 65: 119. 1986. Type: México, Sinaloa, Arroyo de Ibarra, near Rosario 1940, *E*, *Baxter s.n.* (lectotype, designated here, Mammill. Handb.: 169. 1945: Illustration “f. 151 Mammillariaoccidentalisvar.sinalensis X 1”). **Notes.** The protologue indicates that the type specimen was collected, however, it is not mentioned in which herbarium it was deposited. Some specimens collected by Craig (1945) were deposited in the UC herbarium, currently fused with the CAS herbarium. A search was made in the CAS database (https://www.calacademy.org/scientists/botany-collections) and it was not possible to locate the material, on the other hand, type specimens deposited in CAS from UC apparently were lost (Breslin et al. 2021).
= Mammillariamazatlanensisvar.monocentra R.T.Craig, Mammillaria Handb.: 242, 1945. Type: México, Sonora, Yaqui Valley, in the lower delta of the Río Yaqui 1936, *J. Hilton & R. T. Craig s.n*. (lectotype, designated here, Mammill. Handb.: 242. 1945: Illustration “f. 219 Mammillariamazatlanensisvar.monocentra X 1”). **Notes.** See Mammillariaoccidentalisvar.sinalensis

### ﻿*Pelecyphora*

Phylogenetic evidence supports the transference of *Escobaria* to *Pelecyphora* (see discussion) which results in 25 new combinations. Also, nine lectotypes, and three isolectotypes are proposed. Twenty species and 14 subspecies of *Pelecyphora*, are recognized.

#### ﻿ Pelecyphora

Taxon classificationPlantaeCaryophyllalesCactaceae

Ehrenb., Bot. Zeitung (Berlin) 1: 737. 1843.

= Cochiseia W.Earle, Saguaroland Bull. 30: 65. 1976. Type: Cochiseiarobbinsorum W.Earle.
= Encephalocarpus A.Berger, Kakteen 331. 1929. Type: Encephalocarpusstrobiliformis (Werderm.) A.Berger.
= Escocoryphantha Doweld, Sukkulenty 1: 10. 1999. Type: Escocoryphanthachihuahuensis (Britton & Rose) Doweld.
= Escobaria Britton & Rose, Cactaceae 4: 53. 1923. Type: Escobariatuberculosa (Engelm.) Britton & Rose.
= Escobesseya Hester, Desert Pl. Life 17: 23. 1945. Type: Escobesseyadasyacantha (Engelm.) Hester, Desert Pl. Life 17: 25. 1945.
= Fobea Frič ex Boed., Kakteenkunde 1933: 155. 1933. Type: Fobeaviridiflora Frič ex Boed.
= Lepidocoryphantha Backeb., Blätt. Kakteenf. 1938: 22. 1938. Type: Lepidocoryphanthamacromeris (Engelm.) Bakeb.
= Neobesseya Britton & Rose, Cactaceae 4: 51. 1923. Type: Neobesseyamissouriensis (Sweet) Britton & Rose.

##### Type.

*Pelecyphoraaselliformis* Ehrenb.

#### ﻿ Pelecyphora abdita

Taxon classificationPlantaeCaryophyllalesCactaceae

(Řepka & Vaško) D.Aquino & Dan.Sánchez comb. nov.

urn:lsid:ipni.org:names:77248941-1

≡ Escobariaabdita Řepka & Vaško, Cact. Succ. J. (Los Angeles) 83: 265. 2012. Neobesseyaabdita (Řepka & Vaško) Lodé, Cact.-Avent. Int. 98(Suppl.): 6. 2013. Type: México, Coahuila, basin east of the settlement El Oro, 1100 m, Oct 2011, *M. K. Hernández s.n.* (holotype: IZTA).

#### ﻿ Pelecyphora abdita subsp. tenuispina

Taxon classificationPlantaeCaryophyllalesCactaceae

(Pérez-Badillo, Delladdio & Raya-Sánchez) D.Aquino & Dan.Sánchez comb. nov.

urn:lsid:ipni.org:names:77248942-1

≡ Escobariaabdita Řepka & Vaško subsp.tenuispina Pérez-Badillo, Delladdio & Raya-Sánchez, Piante Grasse 36: 9. 2016. Type: México, Coahuila, Parras de la Fuente, *G. B. Hinton 29727* (holotype: GBH).

#### ﻿ Pelecyphora alversonii

Taxon classificationPlantaeCaryophyllalesCactaceae

(J.M.Coult.) D.Aquino & Dan.Sánchez comb. nov.

urn:lsid:ipni.org:names:77248943-1

≡ Cactusradiosusvar.alversonii J.M.Coult. Contr. U.S. Natl. Herb. 3: 122. 1894. Mammillariaalversonii (J.M.Coult.) Zeiss., Monatsschr. Kakteenk. 5: 70. 1895. Mammillariaradiosavar.alversonii (J.M.Coult.) K.Schum., Gesamtbeschr. Kakt.: 481. 1898. Coryphanthaalversonii (J.M.Coult.) Orcutt, Cactography: 3. 1926. Mammillariaviviparavar.alversonii (J.M.Coult.) L.D.Benson, Cacti Ariz: 118. 1950. Coryphanthaviviparavar.alversonii (J.M.Coult.) L.D.Benson, Cacti Ariz. ed. 3: 26. 1969. Escobariaviviparavar.alversonii (J.M.Coult.) D.R.Hunt, Cact. Succ. J. Gr. Brit. 40: 13. 1978. Escobariaalversonii (J.M.Coult.) N.P.Taylor, Cactaceae Consensus Init. 3: 10. 1997. Type: United States, California, Mohave desert Calif., *A. H. Alverson s.n.* (lectotype, designated by Benson Cacti Ariz. 3 ed.: 200. 1969: UC [205017 image!]; isolectotype: F [260000 image!]). **Notes.** The isolectotype label also indicates the date of collection in 1892.

#### ﻿ Pelecyphora aselliformis

Taxon classificationPlantaeCaryophyllalesCactaceae

Ehrenb., Bot. Zeitung 1: 737. 1843.

= Ariocarpusaselliformis (Ehrenb.) F.A.C.Weber, Dict. Hort. 2: 931. 1898. Anhaloniumaselliforme (Ehrenb.) F.A.C.Weber, Dict. Hort. 2: 931. 1898. Type: México, San Luis Potosí, 18 miles [28.96 km] north of San Luis Potosí, 31 Jul 1959, *E. F. Anderson 1206* (neotype, designated by Anderson & Boke, Amer. J. Bot.: 325. 1969: POM [298106]).

#### ﻿ Pelecyphora chihuahuensis

Taxon classificationPlantaeCaryophyllalesCactaceae

(Britton & Rose) D.Aquino & Dan.Sánchez comb. nov.

urn:lsid:ipni.org:names:77248944-1

≡ Escobariachihuahuensis Britton & Rose, Cactaceae 4: 55. 1923. Coryphanthachihuahuensis (Britton & Rose) A.Berger, Kakteen: 339. 1929. Escocoryphanthachihuahuensis (Britton & Rose) Doweld, Sukkulenty 2: 10. 1999. Type: México, Chihuahua, Vicinity of Chihuahua, 08 Apr 1908, *E. Palmer 72* (holotype: US [573550]; isotype: K [250731 image!]).
= Mammillariastrobiliformis Scheer ex Salm-Dyck, Cact. Hort. Dyck. 104–105. 1850, not Mammillariastrobiliformis Engelm., Mem. Tour N. Mexico: 113. 1848, not Mammillariastrobiliformis Muehlenpf., Allg. Gartenzeitung 16: 19. 1848. Echinocactusstrobiliformis Poselg., Allg. Gartenzeitung 21: 107. 1853. Cactusstrobiliformis (Sheer) Kuntze, Revis. Gen. Pl. 1: 261. 1891. Escobariastrobiliformis (Poselg.) F.Boedeker, Mammillarien-Vergleichs-Schlüssel 16. 1933. Coryphanthastrobiliformis (Poselg.) Moran, Gentes Herbarium 8: 318. 1953. Type: [Fragments from] Potts’s original specimen, cult. 1857, hort., Jan 1857, *J.M.F.A.H.I.Salm-Reifferscheid-Dyck s.n.* (lectotype, designated by Benson, Cact. Succ. J. (Los Angeles): 189. 1969: MO). **Notes.**Britton and Rose (1923) chose Engelmann’s epithet *tuberculosa* over *strobiliformis*, because the last represents a homonym. However, Benson (1969) suggested that the epithet *strobiliformis* should be preferred over the epithet *tuberculosa*. Zimmerman and Parfitt (1993+) mention that Escobariatuberculosa and E.strobiliformis represent two independent entities and the name E.chihuahuensis Britton & Rose should be considered a synonym of E.strobiliformis. Given the difference in opinions, Hunt et al. (2006) explained that the name Escobariastrobiliformis has been incorrectly applied to E.tuberculosa and should be rejected. Hunt (2016) concludes that E.strobiliformis is an inadmissible name or with indeterminate application. In order to maintain the stability of the names listed in this treatment, the name Mammillariastrobiliformis is considered a homonym and should not be applied (Turland et al. 2018). In turn, this decision makes it possible to retain the epithet *strobiliformis* for the name Pelecyphorastrobilformis (Werderm.) Fric. & Schelle (basionym Ariocarpusstrobiliformis Werderm.). Finally, original description is not complete and lacks data on floral characters, so it is not feasible to decide on the correct interpretation.
= Mammillariastrobiliformisvar.caespititia Quehl, Monatsschr. Kakteenk. 19: 173. 1909. Mammillariastrobiliformisf.caespititia (Quehl) Schelle, Kakteen: 285. 1926. Escobariatuberculosavar.caespititia (Quehl) Borg, Cacti 304. 1937. Type: Probably Mexico. (Not preserved).

#### ﻿ Pelecyphora chihuahuensis subsp. henricksonii

Taxon classificationPlantaeCaryophyllalesCactaceae

(Glass & R.A.Foster) D.Aquino & Dan.Sánchez comb. nov.

urn:lsid:ipni.org:names:77248945-1

≡ Escobariahenricksonii Glass & R.A.Foster, Cact. Succ. J. (Los Angeles) 49: 195. 1977. Coryphanthahenricksonii (Glass & R.A.Foster) Glass & R.A.Foster, Cact. Succ. J. (Los Angeles) 51: 125. 1979. Escobariachihuahuensissubsp.henricksonii (Glass & R.A.Foster) N.P.Taylor, Cactaceae Consensus Init. 5: 13. 1998. Escocoryphanthahenricksonii (Glass & R.A.Foster) Doweld, Sukkulenty 2: 10. 1999. Type: México, Chihuahua, c. 16 mi. [25.74 km] E of Escalón, Sep 1972, *J. S. Henrickson 7744* (holotype: POM [325439 image, two sheets!]).

#### ﻿ Pelecyphora cubensis

Taxon classificationPlantaeCaryophyllalesCactaceae

(Britton & Rose) D.Aquino & Dan.Sánchez comb. nov.

urn:lsid:ipni.org:names:77248946-1

≡ Coryphanthacubensis Britton & Rose, Torreya 12: 15. 1912. Neobesseyacubensis (Britton & Rose) Hester, Desert Pl. Life 13: 192. 1941. Escobariacubensis (Britton & Rose) D.R.Hunt, Cact. Succ. J. Gr. Brit. 40: 13. 1978. Type: Cuba, Holguín, Oriente, 1909, *J. A. Shafer 2946* (lectotype, designated here: NY [120678 image!]; isolectotype: US [1821121 image!]). **Notes.** According to Britton and Rose (1912), the original specimen of Coryphanthacubensis was kept in cultivation at the New York Botanical Garden. A specimen deposited in NY (120678!) whose data on the label coincide with those referred to in the protologue. Elements such as collector and number (J. A. Shafer 2946) and date of collection (1909) coincide with the label of the specimen referred to here, which is why we designate it as lectotype, while the specimen deposited in the US herbarium (1821121 image!) corresponds to the isolectotype.

#### ﻿ Pelecyphora dasyacantha

Taxon classificationPlantaeCaryophyllalesCactaceae

(Engelm.) D.Aquino & Dan.Sánchez comb. nov.

urn:lsid:ipni.org:names:77248947-1

≡ Mammillariadasyacantha Engelm., Proc. Amer. Acad. Arts 3: 268. 1856. Cactusdasyacanthus (Engelm.) Kuntze, Revis. Gen. Pl. 1: 259. 1891. Coryphanthadasyacantha (Engelm.) Orcutt, Circular to Cactus Fanciers: 1. 1922. Escobariadasyacantha (Engelm.) Britton & Rose, Cactaceae 4: 55. 1923. Escobesseyadasyacantha (Engelm.) Hester, Desert Pl. Life 17: 25. 1945. Neobesseyadasyacantha (Engelm.) Lodé, Cact.-Avent. Int. 98(Suppl.): 6. 2013. Type: United States, Texas, El Paso, 1852, *C. Wright s.n.* (lectotype, designated by Benson, Cacti U. S. Canada: 964. 1982: MO [106919 image!]).
= Mammillariachlorantha Engelm., Rep. U.S. Geogr. Surv., Wheeler 6: 127. 1878. Cactusradiosusvar.chloranthus (Engelm.) J.M.Coult., Contr. U.S. Natl. Herb. 3: 121. 1894. Mammillariaradiosaf.chlorantha (Engelm.) Schelle, Handb. Kakteenkult.: 235. 1907. Coryphanthachlorantha (Engelm.) Britton & Rose, Cactaceae 4: 43. 1923. Mammillariaviviparavar.chlorantha (Engelm.) L.D.Benson, Cacti Ariz., ed. 2 117. 1950. Escobariachlorantha (Engelm.) Buxb., Oesterr. Bot. Z. 98: 78. 1951. Type. United States, St George, May 1874, *C. C. Parry s.n.* (lectotype, designated by Benson, Cacti U. S. Canada: 961. 1982: MO).

#### ﻿ Pelecyphora dasyacantha subsp. chaffeyi

Taxon classificationPlantaeCaryophyllalesCactaceae

(Britton & Rose) D.Aquino & Dan.Sánchez comb. nov.

urn:lsid:ipni.org:names:77248948-1

≡ Escobariachaffeyi Britton & Rose, Cactaceae 4: 56. 1923. Coryphanthachaffeyi (Britton & Rose) Fosberg, Bull. S. Calif. Acad. Sci. 30: 58. 1931. Mammillariachaffeyi (Britton & Rose) Backeb., Neue Kakteen: 16. 1931. Escobariadasyacanthavar.chaffeyi (Britton & Rose) N.P.Taylor, Kakteen And. Sukk. 34: 157. 1983. Escobariadasyacanthasubsp.chaffeyi (Britton & Rose) N.P.Taylor, Cactaceae Consensus Init. 5: 13. 1998. Neobesseyadasyacanthasubsp.chaffeyi (Britton & Rose) Lodé, Cact.-Avent. Int. 98(Suppl.): 6. 2013. Type. México, Zacatecas, near Cedros, Jun 1910, *E. Chaffey 5* (lectotype, designated here: US [1821124 image!]; isolectotype: NY [image 271944!]). **Notes.** A label attached to the specimen deposited in the US (1821124!) indicates that this specimen was designated as a lectotype. However, the typification was not published, so it cannot be accepted (Turland et al. 2018). The sample is part of the original collection since it was collected in the type locality by E. Chaffey, coinciding with the data stipulated in the protologue. Therefore, we formalize the typification of the US specimen.
= Escobariafobei Frič ex A.Berger, Kakteen: 280. 1929. Type. not designated.
= Fobeaviridiflora Frič ex Boed., Kakteenkunde 1933: 155. 1933. Escobariachaffeyif.viridiflora (Frič) Říha, Kaktusy (Brno) 22: 25. 1986. Type: not designated.

#### ﻿ Pelecyphora duncanii

Taxon classificationPlantaeCaryophyllalesCactaceae

(Hester) D.Aquino & Dan.Sánchez comb. nov.

urn:lsid:ipni.org:names:77248949-1

≡ Escobesseyaduncanii Hester, Desert Pl. Life 13: 192. 1941. Escobariaduncanii (Hester) Buxb. Kakteen, Lief. 14, 108c. 1960. Escobariaduncanii (Hester) Backeb., Cactaceae 5: 2966. 1961. Coryphanthaduncanii (Hester) L.D.Benson, Cact. Succ. J. (Los Angeles) 41: 189. 1969. Escobariadasyacanthavar.duncanii (Hester) N.P.Taylor, Kakteen And. Sukk. 34: 157. 1983. Neobesseyaduncanii (Hester) Lodé, Cact.-Avent. Int. 98(Suppl.): 6. 2013. Type: United States, Texas, Brewster County, in the Edwards limestone of mountains a few mi NW of Terlingua in the Edwards limestone, 3400 ft [1036 m], 1937, *F. Duncan s.n.* (holotype: DS [271944]).

#### ﻿ Pelecyphora emskoetteriana

Taxon classificationPlantaeCaryophyllalesCactaceae

(Quehl) D.Aquino & Dan.Sánchez comb. nov.

urn:lsid:ipni.org:names:77248950-1

≡ Mammillariaemskoetteriana Quehl, Monatsschr. Kakteenk. 20: 139. 1910. Coryphanthaemskoetteriana (Quehl) A.Berger, Kakteen: 339. 1929. Escobariaemskoetteriana (Quehl) Borg, Cacti: 304. 1937. Neobesseyaemskoetteriana (Quehl) Lodé, Cact.-Avent. Int. 98(Suppl.): 6. 2013. Type: México, San Luis Potosí, raised in Germany from seed received from San Luís Potosí, *R. Emsköttter s. n.* (lectotype, designated here: US [2767373 image! = Monatsschr. Kakteenk.: 139. 1910. Illustration “Mamillaria Emskötteriana Quehl. Nach einer von Herrn Emil Weddy in Halleaufgenommenen Photographie]). **Notes.** The photograph published in the protologue is considered part of the original material and is designated here as a lectotype (Quehl 1910). A specimen deposited in the US herbarium (2767373!) consists of a duplicate of the original photograph mounted on the sheet. The label indicates it was designated as a lectotype by A. Zimmerman, but it was not published. Here, we formalize this proposal.
= Escobariabella Britton & Rose, Cactaceae 4: 56. 1923. Coryphanthabella (Britton & Rose) Fosberg, Bull. S. Calif. Acad. Sci. 30: 58. 1931. Type. United States, Texas, on hills of Devil’s River, 16 Oct 1913, *J. N. Rose & W. Ficht 17991* (lectotype, designated by Benson, Cacti U. S. Canada: 963. 1982: US [1821125 image!]).
= Escobariarunyonii Britton & Rose, Cactaceae 4: 55. 1923. Mammillariaescobaria Cory, Rhodora 38: 405. 1936. nom. nov. Type. United States, Texas, Rio Grande city, 10 Aug 1921, *R. Runyon s.n.* (lectotype, designated by Benson, Cacti U. S. Canada: 964. 1982: US [not numbered]).
= Coryphantharoberti A.Berger, Kakteen: 280. 1929. Type. United States, Texas, vom Río Grande, Type: not preserved.
= Coryphanthamuehlbaueriana Boed., Monatsschr. Deutsch. Kakteen-Ges. 2: 18. 1930. Escobariamuehlbaueriana (Boed.) F.M.Knuth, Kaktus-ABC: 380. 1936. Neobesseyamuehlbaueriana (Boed.) Boed., Mammill.-Vergl.-Schluessel: 15. 1933. Type. México, Tamaulipas, bei Jaumave, 1929, *F. Viereck s.n.* (lectotype, designated here, Monatsschr. Deutsch. Kakteen-Ges.: 18. 1930a: Illustration “Coryphanthamuehlbaueriana Boed. sp. nov. Nat. Gr.”).

#### ﻿ Pelecyphora hesteri

Taxon classificationPlantaeCaryophyllalesCactaceae

(Y.Wright) D.Aquino & Dan.Sánchez comb. nov.

urn:lsid:ipni.org:names:77248951-1

≡ Coryphanthahesteri Y.Wright, Cact. Succ. J. (Los Angeles) 4: 274. 1932. Escobariahesteri (Y.Wright) Buxb., Oesterr. Bot. Z. 98: 78. 1951. Type: United States, Hill on U.S. 385, 3.5 miles south of U.S. 90 east of Marathon. South side of gap and road cut. Crest of hill. Drainage Area Rio Grande, 06 Apr 1965, *L. D. Benson & B. H. Warnock*, *16500* (neotype, designated by Benson, Cacti U. S. Canada: 961. 1982: POM [315706 image!]).

#### ﻿ Pelecyphora hesteri subsp. grata

Taxon classificationPlantaeCaryophyllalesCactaceae

(Kaplan, Kunte & Snicer) D.Aquino & Dan.Sánchez comb. nov.

urn:lsid:ipni.org:names:77248952-1

≡ Escobariagrata Kaplan, Kunte & Snicer, Kaktusy (Brno) 37: 37. 2001. Escobariahesterisubsp.grata (Kaplan, Kunte & Snicer) Lüthy & Dicht, Cact. World 25: 175. 2007. Type: México, Coahuila, collibus calcareis montibus Sierra el Burro ca 150 km situ septentrio-occidentali ab oppido Monclova, *J. Snicer et al. s.n.* (holotype: PR).

#### ﻿ Pelecyphora laredoi

Taxon classificationPlantaeCaryophyllalesCactaceae

(Glass & R.A.Foster) D.Aquino & Dan.Sánchez comb. nov.

urn:lsid:ipni.org:names:77248953-1

≡ Coryphanthalaredoi Glass & R.A.Foster, Cact. Succ. J. (Los Angeles) 50: 235. 1978. Escobarialaredoi (Glass & R.A.Foster) N.P.Taylor, Cact. Succ. J. Gr. Brit. 41: 20. 1979. Type: México, Coahuila, SE Coahuila, about 2 km N of El Cinco, SE of General Cepeda, near top of a mountain pass, Sierra de Parras, Feb 1972, *C. Glass & R. Foster 3761* (holotype: POM; isotype: ASU [0018460 image!]).

#### ﻿ Pelecyphora lloydii

Taxon classificationPlantaeCaryophyllalesCactaceae

(Britton & Rose) D.Aquino & Dan.Sánchez comb. nov.

urn:lsid:ipni.org:names:77248954-1

≡ Escobarialloydii Britton & Rose, Cactaceae 4: 57. 1923. Coryphanthalloydii (Britton & Rose) Fosberg, Bull. S. Calif. Acad. Sci. 30: 58. 1931. Neobesseyalloydii (Britton & Rose) Lodé, Cact.-Avent. Int. 98(Suppl.): 7. 2013. Type: México, Zacatecas, Foothills of Sra. Zuluago [Sierra de Zuloaga], 29 Mar 1908, *F.E. Lloyd 5* (holotype: US [535108 image!]).

#### ﻿ Pelecyphora macromeris

Taxon classificationPlantaeCaryophyllalesCactaceae

(Engelm.) D. Aquino & Dan.Sánchez comb. nov.

urn:lsid:ipni.org:names:77248955-1

≡ Mammillariamacromeris Engelm. Mem. Tour N. Mexico [Wislizenus] 97. 1848. Echinocactusmacromeris (Engelm.) Poselg. Allg. Gartenzeitung 21: 102. 1853. Coryphanthamacromeris (Engelm.) Lem., Cactées 35. 1868. Lepidocoryphanthamacromeris (Engelm.) Backeb. Cactaceae (Berlin) 1941: 61. 1942. Type: United States, New México, sandy soil near Doñana [Dona Ana], 08 May 1846, *A. Wislizenius s.n*. (lectotype, designated by Benson, Cacti U. S. Canada: 959. 1982: MO [2017406 image!, 2017407 image!, two sheets]).
= Mammillariadactylothele Labour., Monogr. Cact.: 146. 1853. Type. Not designated.

#### ﻿ Pelecyphora macromeris subsp. runyonii

Taxon classificationPlantaeCaryophyllalesCactaceae

(Britton & Rose) D.Aquino & Dan.Sánchez comb. nov.

urn:lsid:ipni.org:names:77248956-1

≡ Coryphantharunyonii Britton & Rose, Cactaceae (Britton & Rose) 4: 26. 1923. Lepidocoryphantharunyonii (Britton & Rose) Backeb., Cactaceae (Backeberg) 5: 2975. 1961. Coryphanthamacromerisvar.runyonii (Britton & Rose) L.D.Benson, Cact. Succ. J. (Los Angeles) 41: 188. 1969. Coryphanthamacromerissubsp.runyonii (Britton & Rose) N.P.Taylor, Cactaceae Consensus Init. 6: 15. 1998. Lepidocoryphanthamacromerissubsp.runyonii (Britton & Rose) Doweld, Sukkulenty 2: 28. 1999. Type: United States, Texas, to Rio Grande [City], 10 Aug 1921, *R. Runyon 15* (lectotype, designated by Benson, Cact. Succ. J. (Los Angeles): 188. 1969: US [2761309 image!]).
= Coryphanthapirtlei Werderm. Notizbl. Bot. Gart. Berlin-Dahlem 12: 226. 1934. Type: United States, Texas, Starr County, 1931. *W. A. Pirtle s.n*. **Notes.**Benson (1982) indicates that the material type of Coryphanthapirtlei was preserved. However, there is no certainty about the herbarium where it was deposited.

#### ﻿ Pelecyphora minima

Taxon classificationPlantaeCaryophyllalesCactaceae

(Baird) D.Aquino & Dan.Sánchez comb. nov.

urn:lsid:ipni.org:names:77248957-1

≡ Coryphanthaminima Baird, Amer. Bot. (Binghamton) 37: 150. 1931. Escobariaminima (Baird) D.R.Hunt, Cact. Succ. J. Gr. Brit. 40: 30. 1978. Neobesseyaminima (Baird) Lodé, Cact.-Avent. Int. 98(Suppl.): 7. 2013. Type: United States, Texas, near Marathon, Mar 1931, *A. R. Davis s.n* (lectotype, designated by Benson, Cacti U. S. Canada: 959. 1982: US [1530466 image!]).
= Coryphanthanellieae Croizat, Torreya 34: 15. 1934. Escobarianellieae (Croizat) Backeb., Cactaceae 5: 2967. 1961. Mammillarianellieae (Croizat) Croizat, Cact. Succ. J. (Los Angeles) 14: 34. 1942. Type. United States, Texas, about 4 miles south of Marathon, in limestone formations, *Davis s.n.* (holotype: NY).

#### ﻿ Pelecyphora missouriensis

Taxon classificationPlantaeCaryophyllalesCactaceae

(Sweet) D.Aquino & Dan.Sánchez comb. nov.

urn:lsid:ipni.org:names:77248958-1

≡ Mammillariamissouriensis Sweet, Hort. Brit.: 171. 1826. Cactusmissouriensis (Sweet) Kuntze, Revis. Gen. Pl. 1: 259. 1891. Mammillariamissouriensis Sweet ex K.Schum., Gesamtbeschr. Kakt.: 498. 1898. Coryphanthamissouriensis (Sweet) Britton & Rose, Ill. Fl. N. U.S. 2: 570. 1913. Neobesseyamissouriensis (Sweet) Britton & Rose, Cactaceae 4: 53. 1923. Neomammillariamissouriensis (Sweet) Britton & Rose ex Rydb., Fl. Plains N. Amer. 561. 1932. Escobariamissouriensis (Sweet) D.R.Hunt, Cact. Succ. J. Gr. Brit. 40: 13. 1978. Type: United States, North Dakota, Burleigh County, 3 mi [4.8 km] W of Baldwin turnoff, 1.3 m [2.09 km] E of Missouri River, Jun 1970, *L. Mitich s.n.* (neotype, designated by Mitich & Benson, Cact. Succ. J. (Los Angeles): 8. 1977: POM [317949]; isoneotype NDA).
= Mammillariasimilis Engelm. & A.Gray, Boston J. Nat. Hist. 5: 246. 1845. Echinocactussimilis (Engelm.) Poselg., Allg. Gartenzeitung 21: 107. 1853. Cactusmissouriensisvar.similis (Engelm.) J.M.Coult. in Contr. U.S. Natl. Herb. 3: 111. 1894. Cactussimilis (Engelm.) Small, Fl. S.E. U.S.: 812. 1903. Coryphanthasimilis (Engelm.) Britton & Rose, Ill. Fl. N. U.S.: 571. 1913. Neobesseyasimilis (Engelm.) Britton & Rose, Cactaceae 4: 52. 1923. Escobariamissouriensisvar.similis (Engelm.) N.P.Taylor, Kakteen And. Sukk. 34: 184. 1983. Neobesseyamissouriensissubsp.similis (Engelm.) Doweld, Sukkulenty 3: 37. 2000. Type: United States, Sandstone rocks, near Industry (not preserved).
= Mammillarianuttallii Engelm., Mem. Amer. Acad. Arts n.s., 4: 49. 1849. Coryphanthanuttallii Engelm. ex C.F.Först., Handb. Cacteenk.: 407. 1885. Mammillariamissouriensisvar.nuttallii (Engelm.) Schelle, Handb. Kakteenkult.: 241. 1907. Neobesseyanuttallii Boed., Mammill.-Vergl.-Schluessel: 15. 1933. Neobesseyanuttallii (Engelm.) Borg, Cacti: 303. 1937. Type. United States, South Dakota, Ft. Pierre, on the Upper Missouri, 1847, *F. V. Hayden s.n.* (lectotype, designated by Benson, Cacti U. S. Canada: 964. 1982: MO [899104 image!, 899105 image!, two sheets]).
= Mammillariasimilisvar.robustior Engelm., Boston J. Nat. Hist. 6: 200. 1850. Mammillarianuttalliivar.robustior (Engelm.) Engelm. & J.M.Bigelow, Pacif. Railr. Rep. 4: 28. 1856. Mammillariamissouriensisvar.robustior (Engelm.) S.Watson, Bibl. Index N. Amer. Bot.: 440. 1878. Cactusmissouriensisvar.robustior (Engelm.) J.M.Coult., Contr. U.S. Natl. Herb. 3: 111. 1894. Coryphanthamissouriensisvar.robustior (Engelm.) L.D.Benson, Cact. Succ. J. (Los Angeles) 41: 190. 1969. Escobariamissouriensisvar.robustior (Engelm.) D.R.Hunt, Cact. Succ. J. Gr. Brit. 40: 13. 1978. Type: United States, Texas, Piedernales [Perdenales] [River, Texas], May 1846, *F. Lindheimer s.n*. (lectotype, designated by Benson, Cact. Succ. J. (Los Angeles): 190. 1969: MO [2017430 image!]).
= Mammillariasimilisvar.caespitosa Engelm., Boston J. Nat. Hist. 6: 200. 1850. Mammillarianuttalliivar.caespitosa Engelm., Proc. Amer. Acad. Arts 3: 265. 1856. Mammillariamissouriensisvar.caespitosa (Engelm.) S.Watson, Smithsonian Misc. Collect. 258: 403. 1878. Mammillariawissmannii Hildm. ex K.Schum., Gesamtbeschr. Kakt.: 498. 1898. nom. nov.Neobesseyawissmannii (Hildm. ex K.Schum.) Britton & Rose, Cactaceae 4: 52. 1923. Coryphanthawissmannii (Hildm. ex K.Schum.) A.Berger, Kakteen: 278. 1929. Coryphanthamissouriensisvar.caespitosa (Engelm.) L.D.Benson, Cact. Succ. J. (Los Angeles) 41: 189. 1969. Escobariamissouriensisvar.caespitosa (Engelm.) D.R.Hunt, Cact. Succ. J. Gr. Brit. 40: 13. 1978. Type: United States, Cult. In hort. Göbels, St Louis from Texas near Industry, May 1846, *F. Lindheimer s.n*. (lectotype, designated by Benson, Cact. Succ. J. (Los Angeles): 190. 1969: MO).
= Mammillarianuttalliivar.borealis Engelm., Proc. Amer. Acad. Arts 3: 264. 1856. Type: United States, on the Upper Missouri. Not preserved.
= Mammillarianotesteinii Britton, Bull. Torrey Bot. Club 18: 367. 1891. Cactusnotesteinii (Britton) Rydb., Mem. New York Bot. Gard. 1: 272. 1900. Neobesseyanotesteinii (Britton) Britton & Rose, Cactaceae 4: 53. 1923. Type: United States, Deer Londge, Mont., 01 Jun 1891, *F. N. Notestein s.n*. (lectotype, designated here: NY [385874 image, four sheets!]; isolectotype: US [1821122 image!]).
= Coryphanthamarstonii Clover, Bull. Torrey Bot. Club 65: 412. 1938. Coryphanthamissouriensisvar.marstonii (Clover) L.D.Benson, Cacti Ariz. ed. 3: 26. 1969. Escobariamissouriensisvar.marstonii (Clover) D.R.Hunt, Cact. Succ. J. Gr. Brit. 40: 13. 1978. Type. United States, Utah, Kane County, east side of Buckskin Mountains, 5200 ft [1584 m], 08 Aug 1953, *L. D. Benson & R. Benson 15205* (neotype, designated by Benson, Cacti Ariz. ed. 3: 26. 1969: POM [285320, 296309, two sheets]).
= Escobariamissouriensissubsp.navajoensis Hochstätter, Succulenta (Netherlands) 75: 257. 1996. Type. United States, Arizona, Navajoa, 1600–1800, *F. Hochstätter 1000* (holotype: HBG).

#### ﻿ Pelecyphora missouriensis subsp. asperispina

Taxon classificationPlantaeCaryophyllalesCactaceae

(Boed.) D.Aquino & Dan.Sánchez comb. nov.

urn:lsid:ipni.org:names:77248959-1

≡ Coryphanthaasperispina Boed., Monatsschr. Deutsch. Kakteen-Ges. 1: 192. 1929. Neobesseyaasperispina (Boed.) Boed., Mammill.-Vergl.-Schluessel: 14. 1933. Neobesseyaasperispina (Boed.) Boed. ex Backeb. & F.M.Knuth, Kaktus-ABC: 379. 1936. Escobariaasperispina (Boed.) D.R.Hunt, Cact. Succ. J. Gr. Brit. 40: 13. 1978. Escobariamissouriensisvar.asperispina (Boed.) N.P.Taylor, Kakteen And. Sukk. 34: 185. 1983. Escobariamissouriensissubsp.asperispina (Boed.) N.P.Taylor, Cactaceae Consensus Init. 5: 13. 1998. Neobesseyamissouriensissubsp.asperispina (Boed.) Lodé, Cact.-Avent. Int. 100: 30. 2013. Type: Mexico, Coahuila, südlich von Saltillo, und dort in grasigen, 2500 m, *F. Ritter s.n.* (lectotype, designated here, Monatsschr. Deutsch. Kakteen-Ges.: 192. 1929: Illustration “Coryphanthaasperispina Boed. sp. nov. natür. Gröβe”).

#### ﻿ Pelecyphora robbinsorum

Taxon classificationPlantaeCaryophyllalesCactaceae

(W.H.Earle) D.Aquino & Dan.Sánchez comb. nov.

urn:lsid:ipni.org:names:77248960-1

≡ Cochiseiarobbinsorum W.H.Earle, Saguaroland Bull. 30: 65. 1976. Coryphantharobbinsorum (W.H.Earle) A.D.Zimmerman, Cact. Succ. J. (Los Angeles) 50: 294. 1978. Escobariarobbinsorum (W.H.Earle) D.R.Hunt, Cact. Succ. J. Gr. Brit. 40: 13. 1978. Neobesseyarobbinsorum (W.H.Earle) Doweld, Sukkulenty 3: 37. 2000. Type: United States, Arizona, SE Cochise County, rocky hills, 4250 ft [1280 m], 1976, *J. Robbins et al. s.n.* (holotype: ASU [18455]).

#### ﻿ Pelecyphora sneedii

Taxon classificationPlantaeCaryophyllalesCactaceae

(Britton & Rose) D.Aquino & Dan.Sánchez comb. nov.

urn:lsid:ipni.org:names:77248961-1

≡ Escobariasneedii Britton & Rose, Cactaceae 4: 56. 1923. Coryphanthasneedii (Britton & Rose) A.Berger, Kakteen: 280. 1929. Mammillariasneedii (Britton & Rose) Cory, Rhodora 38: 407. 1936. Type: United States, Texas, 8 km N of El Paso, McKelligan Canyon, Mt. Franklin., 22 Feb 1921, *S. L. Pattinson s.n*. (lectotype, designated by Benson, Cacti U. S. Canada: 963. 1969: US [image 2767376!]).
= Escobariaalbicolumnaria Hester, Desert Pl. Life 13: 129. 1941. Coryphanthaalbicolumnaria (Hester) Zimmerman, Cact. Succ. J. (Los Angeles) 44: 157. 1972. Escobariasneediisubsp.albicolumnaria (Hester) Lüthy, Kakteen And. Sukk. 50: 278. 1999. Coryphanthasneediivar.albicolumnaria (Hester) A.D.Zimmerman, Cacti Trans-Pecos: 424. 2004. Type: United States, Texas, mountainous limestone area W.N.W. of Terlingua and N.E. of Lajitas, in the southern part of Brewster County, 01 Apr 1940, *J. P. Hester s.n.* (holotype: DS [271855 image!, two sheets]).
= Escobariaguadalupensis S.Brack & K.D.Heil, Cact. Succ. J. (Los Angeles) 58: 165. 1986. Coryphanthasneediivar.guadalupensis (S.Brack & K.D.Heil) A.D.Zimmerman, Cacti Trans-Pecos: 420. 2004. Type: United States, Texas, Culberson County, Guadalupe Mountains National Park, 2000–2600 m, *Heil et al. s. n.* (holotype: SJNM).
= Escobarialeei Rose ex Boed., Mammillarien-Vergleichs-Schluessel: 17. 1933. Coryphanthasneediivar.leei (Rose) L.D.Benson, Cact. Succ. J. (Los Angeles) 41: 189. 1969. Escobariasneediivar.leei (Rose ex Boed.) D.R.Hunt, Cact. Succ. J. Gr. Brit. 40: 30. 1978. Escobariasneediisubsp.leei (Rose ex Boed.) D.R.Hunt, Cactaceae Consensus Init. 4: 5. 1997. Type: United States, New México, Rattlesnake Canyon, 30 mi SW of Carlsbad, 5500 ft [1676 m] 1924, *W.T. Lee s.n.* (lectotype, designated by Castteter & Pierce, Madroño: 138. 1966: US [72134 image!]).

#### ﻿ Pelecyphora sneedii subsp. orcuttii

Taxon classificationPlantaeCaryophyllalesCactaceae

(Boed.) D.Aquino & Dan.Sánchez comb. nov.

urn:lsid:ipni.org:names:77248962-1

≡ Escobariaorcuttii Boed., Mammillarien-Vergleichs-Schluessel: 17. 1933. Escobariasneediisubsp.orcuttii (Boed.) Lüthy, Kakteen And. Sukk. 50: 278. 1999. Coryphanthasneediivar.orcuttii (Boed.) Gorelick, J. Bot. Res. Inst. Texas 9: 28. 2015. Escobariasneediivar.orcuttii (Boed.) Gorelick, J. Bot. Res. Inst. Texas 9: 28. 2015. Type: United States, New Mexico, Granite Pass, Mar 1926, *C. R. Orcutt s.n.* (lectotype, designated by Benson, Cacti Ariz. ed. 3: 26. 1969: DS [307410 image!]).
= Escobariaorcuttii Rose ex Orcutt, Cactography 5, 1926. Nom. inval. Coryphanthaorcuttii (Rose ex Orcutt) Zimmerman, Cact. Succ. J. (Los Angeles) 44: 156. 1972. Nom. inval. Coryphanthastrobiliformisvar.orcuttii (Rose ex Orcutt) L.D.Benson, Cacti Ariz. ed. 3: 156. 1972. nom. inval.
= Escobariaorcuttiivar.koenigii Castetter, P.Pierce & K.H.Schwer., Cact. Succ. J. (Los Angeles) 47: 68. 1975. Type: United States, New México, Luna County, Florida Mts., Central Valley on east slope of the Koenig Ranch on black limestone, E & NE slopes of hill (el.5200 [152.4 m]) which is 500 ft [152.4 m] above plains, 5200 ft [1584 m] 07 May 1962, *E. F. Castetter 961* (holotype: UNM [38768 image!]).
= Escobariaorcuttiivar.macraxina Castetter, P.Pierce & K.H.Schwer., Cact. Succ. J. (Los Angeles) 47: 66. 1975. Type: United States, New México, Hidalgo County, Big Hatchet Mountains, west slope, 21 Dec 1973, *K. D. Heil 4287* (holotype: UNM [54141 image!]; isotypes: UNM [54138 image!, 54142 image!, 54143 image!]).
= Coryphanthaorganensis Zimmerman, Cact. Succ. J. (Los Angeles) 44: 114. 1972. Escobariaorganensis (Zimmerman) Castetter, P.Pierce & K.H.Schwer., Cact. Succ. J. (Los Angeles) 47: 60. 1975. Escobariasneediisubsp.organensis (Zimmerman) Lüthy, Kakteen And. Sukk. 50: 278. 1999. Type: United States, New México, Dona Ana County, c. 15 mi E of Las Cruces, Organ Mountains, 17 Jan 1971, *D. A. Zimmerman & A. D. Zimmerman*, *1535* (holotype: SNM; isotype: DS [642362 image!], MICH [1123478 image!]).
= Escobariasandbergii Castetter, P.Pierce & K.H.Schwer., Cact. Succ. J. (Los Angeles) 47: 62. 1975. Escobariasneediisubsp.sandbergii (Castetter, P.Pierce & K.H.Schwer.) Lüthy, Kakteen And. Sukk. 50: 278. 1999. Type: United States, New México, Sierra County, at Rope Springs, west slope of the San Andres Mts., 01 Apr 1967, *P. Pierce 3409* (holotype: UNM [38739 image!]).
= Escobariavillardii Castetter, P.Pierce & K.H.Schwer., Cact. Succ. J. (Los Angeles) 47: 64. 1975. Escobariasneediisubsp.villardii (Castetter, P.Pierce & K.H.Schwer.) Lüthy, Kakteen And. Sukk. 50: 278. 1999. Type: United States, New México, Otero County, Alamo Canyon, near Alamagordo, 18 Mar 1972, *R. Reeves 3984* (holotype: UNM [50789 image!]).

#### ﻿ Pelecyphora strobiliformis

Taxon classificationPlantaeCaryophyllalesCactaceae

(Werderm.) Fric. & Schelle, Verzeichniss 9, 1935.

≡ Ariocarpusstrobiliformis Werderm., Z. Sukkulentenk. 3: 126. 1927. Encephalocarpusstrobiliformis (Werderm.) A.Berger, Kakteen: 332. 1929. Type: México, Tamaulipas, near Miquihuana, 22 Jan 1961 (neotype, designated by Anderson & Boke, Amer. J. Bot.: 325. 1969: POM [298105]).

#### ﻿ Pelecyphora tuberculosa

Taxon classificationPlantaeCaryophyllalesCactaceae

(Engelm.) D.Aquino & Dan.Sánchez comb. nov.

urn:lsid:ipni.org:names:77248963-1

≡ Mammillariatuberculosa Engelm., Proc. Amer. Acad. Arts 3: 268. 1856. Coryphanthatuberculosa (Engelm.) Orcutt, Circular to Cactus Fanciers: i. 1922. Escobariatuberculosa (Engelm.) Britton & Rose, Cactaceae 4: 54. 1923. Coryphanthatuberculosa (Engelm.) A.Berger, Kakteen: 280. 1929. Type: [México], Flounce mountains below El Paso, Below San Elisario on the Río Grande, Jun 1852, *J. Bigelow s.n.* (lectotype, designated by Benson, Cacti U. S. Canada: 962. 1982: MO [2017442 image!]).
= Mammillariatuberculata Engelm., Syn. Cact. U.S.: 12. 1856. Cactustuberculosus (Engelm.) Kuntze, Revis. Gen. Pl. 1: 261. 1891. **Notes.** When comparing the original description Mammillariatuberculata (https://www.biodiversitylibrary.org/page/32558530#page/12/mode/1up), it clearly corresponds a duplicate of the description of M.tuberculosa Engelm. Therefore, in the absence of diagnosis and designation of a type, it should be considered as *nomen nudum* (Turland et al. 2018).
= Mammillariastrobiliformisvar.rufispina Quehl, Monatsschr. Kakteenk. 17: 87. 1907. Mammillariastrobiliformisf.rufispina (Quehl) Schelle, Kakteen: 285. 1926. Type: Mexico (Not preserved).
= Mammillariastrobiliformisvar.pubescens Quehl, Monatsschr. Kakteenk. 17: 87. 1907. Mammillariastrobiliformisf.pubescens (Quehl) Schelle, Kakteen: 285. 1926. Escobariatuberculosavar.pubescens (Quehl) Y.Itô, Cacti 1952: 113. 1952. Type: Mexico (Not preserved).
= Mammillariastrobiliformisvar.durispina Quehl, Monatsschr. Kakteenk. 17: 87. 1907. Mammillariastrobiliformisf.durispina (Quehl) Schelle, Kakteen: 285. 1926. Escobariatuberculosavar.durispina (Quehl) Børgesen, Borg, J., Cacti 304. 1937. Coryphanthastrobiliformisvar.durispina (Quehl) L.D.Benson, Cact. Succ. J. (Los Angeles) 41: 189. 1969. Escobariastrobiliformisvar.durispina (Quehl) Bravo, Cact. Suc. Mex. 27: 17. 1982. Type. United States, Texas, Brewster County, Terlingua *H. Kuenzler s.n.* (neotype, designated by Benson, Cact. Succ. J. (Los Angeles): 189. 1969: POM [311333 image!]).
= Coryphanthavaricolor Tiegel, Monatsschr. Deutsch. Kakteen-Ges. 3: 278. 1932. Coryphanthadasyacanthavar.varicolor (Tiegel) L.D.Benson, Cact. Succ. J. (Los Angeles) 41: 189. 1969. Escobariadasyacanthavar.varicolor (Tiegel) D.R.Hunt, Cact. Succ. J. Gr. Brit. 40: 13. 1978. Escobariatuberculosavar.varicolor (Tiegel) S.Brack & K.D.Heil, Cact. Succ. J. (Los Angeles) 60: 17. 1988. Escobariatuberculosasubsp.varicolor (Tiegel) Lüthy, Kakteen And. Sukk. 50: 257. 1999. Coryphanthatuberculosavar.varicolor (Tiegel) A.D.Zimmerman, Cacti Trans-Pecos: 436. 2004. Type. United States, Texas, Brewster County, hills south of Marathon, 3800 ft [1158 m],03 Apr 1947, *B. H. Warnock 47–467* (neotype, designated by Benson, Cact. Succ. J. (Los Angeles): 189. 1969: SRSC).
= Escobariastrobiliformissubsp.sisperai Halda & Sladk. Acta Mus. Richnov. Sect. Nat. 7: 35. 2000. Type: México, Nuevo Léon, via bitumine constrata inter-Monterrey et Tampico, non procul a via publica prope compitum Marin, 07 Apr 1985, *J.J. Halda & J. Sladkovský 85040073* (holotype PR).

#### ﻿ Pelecyphora vivipara

Taxon classificationPlantaeCaryophyllalesCactaceae

(Nutt.) D.Aquino & Dan.Sánchez comb. nov.

urn:lsid:ipni.org:names:77248964-1

≡ Cactusviviparus Nutt. Nutt., Cat. Pl. Upper Louisiana no. 22. 1813. Mammillariavivipara (Nutt.) Haw., Suppl. Pl. Succ.: 72. 1819. Echinocactusviviparus Poselg., Allg. Gartenzeitung 21: 107. 1853. Mammillariaradiosavar.vivipara (Nutt.) Schelle, Handb. Kakteenkult.: 236. 1907. Coryphanthavivipara (Nutt.) Britton & Rose, Ill. Fl. N. U.S.: 571. 1913. Escobariavivipara (Nutt.) Buxb., Oesterr. Bot. Z. 98: 78. 1951. Coryphanthaneovivipara Y.Itô, Cactaceae: 556. 1981. comb. inval. Type: United States, North Dakota, McClean County, 12 mi [19.31 km] E of Fort Mandan, E of Missouri River, Jun 1971, *L. Mitich s.n.* (neotype, designated by Mitich & Benson, Cact. Succ. J. (Los Angeles): 8. 1977: POM [317948]; isoneotype NDA).
= Mammillariaradiosa Engelm., Boston J. Nat. Hist. 6: 196. 1850. Echinocactusradiosus Poselg., Allg. Gartenzeitung 21: 107. 1853. Mammillariaviviparavar.radiosa Engelm. Proc. Amer. Acad. Arts 3: 269. 1856. Mammillariaviviparasubsp.radiosa Engelm., Rep. U.S. Mex. Bound. Cact.: 15. 1858. Cactusradiosus (Engelm.) J.M.Coult., Contr. U.S. Natl. Herb. 3: 120. 1894. Coryphantharadiosa (Engelm.) Rydb., Fl. Rocky Mts.: 581. 1917. Neomammillariaradiosa (Engelm.) Rydb., Fl. Plains N. Amer.: 562. 1932. Coryphanthaviviparavar.radiosa (Engelm.) Backeb., Cactaceae 5: 2998. 1961. Escobariaviviparavar.radiosa (Engelm.) D.R.Hunt, Cact. Succ. J. Gr. Brit. 40: 13. 1978. Escobariaradiosa (Engelm.) G.Frank place of publication unknown, nom. inval. Coryphanthaneoviviparavar.radiosa (Engelm.) Y.Itô, Cactaceae: 556. 1981. Type: United States, Texas, sterile soils on the Pierdenales [Pedernales], and cult. In St Louis, Jun 1846, *F. Lindheimer s.n.* (lectotype, designated by Benson, Cacti U. S. Canada: 960. 1982: MO [2017377 image!, 2017376 image!]).
= Mammillariaviviparavar.vera Engelm., Proc. Amer. Acad. Arts 3: 269. 1856. Type: United States. On the upper Missouri and Yellowstone rivers (Not preserved).
= Mammillariaviviparavar.radiosasubvar.neomexicana Engelm., Proc. Amer. Acad. Arts 3: 269. 1856. Cactusradiosusvar.neomexicanus (Engelm.) J.M.Coult., Contr. U.S. Natl. Herb. 3: 120. 1894. Cactusneomexicanus (Engelm.) Small, Fl. S.E. U.S.: 812. 1903. Mammillarianeomexicana (Engelm.) A.Nelson, New Man. Bot. Centr. Rocky Mt.: 327. 1909. Coryphanthaneomexicana (Engelm.) Britton & Rose, Cactaceae 4: 45. 1923. Escobarianeomexicana (Engelm.) Buxb., Oesterr. Bot. Z. 98: 78. 1951. Coryphanthaviviparavar.neomexicana (Engelm.) Backeb., Cactaceae 5: 2999. 1961. Coryphanthaneoviviparavar.neomexicana (Engelm.) Y.Itô, Cactaceae: 556. 1981. nom. inval. Escobariaviviparavar.neomexicana (Engelm.) Buxb., Kakteen (H. Krainz) 108c, 1973. Type: United States, South New Mexico, 1849, *C. Wright s. n*. (lectotype, designated by Benson, Cacti U. S. Canada: 960. 1982: MO [2019650 image!]).
= Mammillariaarizonica Engelm., Bot. California 1: 244. 1876. Cactusradiosusvar.arizonicus (Engelm.) J.M.Coult., Contr. U.S. Natl. Herb. 3: 121. 1894. Mammillariaradiosavar.arizonica (Engelm.) K.Schum., Gesamtbeschr. Kakt.: 481. 1898. Mammillariaradiosaf.arizonica (Engelm.) Schelle, Handb. Kakteenkult.: 235. 1907. Coryphanthaarizonica (Engelm.) Britton & Rose, Cactaceae 4: 45. 1923. Mammillariaviviparavar.arizonica (Engelm.) L.D.Benson, Proc. Calif. Acad. Sci., ser. 4, 25: 263. 1944. Coryphanthaviviparavar.arizonica (Engelm.) W.T.Marshall, Desert. Bot. Gard. Arizona, Sci. Bull. 1: 94. 1950. Escobariaarizonica (Engelm.) Buxb. in Oesterr. Bot. Z. 98: 78. 1951. Escobariaviviparavar.arizonica (Engelm.) D.R.Hunt, Cact. Succ. J. Gr. Brit. 40: 13. 1978. Coryphanthaneoviviparavar.arizonica (Engelm.) Y.Itô, Cactaceae: 556. 1981. Nom. inval. Type: United States, Arizona, *E. Coues & E. Palmer s. n*. (lectotype, designated by Benson, Cacti U. S. Canada: 961. 1982: MO [2017352 image]).
= Mammillariadeserti Engelm., Bot. California 2: 449. 1880. Cactusradiosusvar.deserti (Engelm.) J.M.Coult., Contr. U.S. Natl. Herb. 3: 121. 1894. Mammillariaradiosavar.deserti (Engelm.) K.Schum., Gesamtbeschr. Kakt.: 481. 1898. Mammillariaradiosaf.deserti (Engelm.) Schelle, Handb. Kakteenkult.: 236. 1907. Coryphanthadeserti (Engelm.) Britton & Rose, Cactaceae 4: 46. 1923. Mammillariaviviparavar.deserti (Engelm.) L.D.Benson in Proc. Calif. Acad. Sci., ser. 4, 25: 263. 1944. Coryphanthaviviparavar.deserti (Engelm.) W.T.Marshall, Desert. Bot. Gard. Arizona, Sci. Bull. 1: 94. 1950. Escobariadeserti (Engelm.) Buxb., Oesterr. Bot. Z. 98: 78. 1951. Coryphanthachloranthavar.deserti (Engelm.) Backeb., Cactaceae 5: 3003. 1961. Escobariaviviparavar.deserti (Engelm.) D.R.Hunt, Cact. Succ. J. Gr. Brit. 40: 13. 1978. Type: United States, California, at Ivapah, 30 miles northeast of San Bernardino, in one mountain range stretching into the desert, *S. B. Parish 455* (lectotype, designated by Benson, Cacti U. S. Canada: 961. 1982: MO [2267169 image!]).
= Mammillariahirschtiana F.Haage, Monatsschr. Kakteenk. 6: 127. 1896. Type: No designated.
= Mammillariaradiosavar.texensis Schelle, Handb. Kakteenkult.: 236. 1907. Type: No designated.
= Mammillariaramosissima Quehl, Monatsschr. Kakteenk. 18: 127. 1908. Type: United States, California, *R. C. Orcutt s. n.* (lectotype, designated here, Monatsschr. Kakteenk.: 127. 1908: Illustration “Mamillariaramosissima Quehl Nach einer von Herr De Laet aufgenommenen Photographie”).
= Coryphanthabisbeeana Orcutt, Cactography: 3. 1926. Escobariabisbeeana (Orcutt) Borg, Cacti: 305. 1937. Coryphanthaviviparavar.bisbeeana (Orcutt) L.D.Benson, Cacti Ariz. ed. 3: 25. 1969. Escobariaviviparavar.bisbeeana (Orcutt) D.R.Hunt, Cact. Succ. J. Gr. Brit. 40: 13. 1978. Type: United States, Arizona, *J. N. Rose 11958* (lectotype, designated by Benson, Cacti Ariz. ed 3: 25. 1969: US [3050430 image!]).
= Coryphanthacolumnaris Lahman, Cact. Succ. J. (Los Angeles) 6: 27. 1934. Type: United States, Oklahoma, Jackson County, near Altus, 600 ft [182 m], 1926, *M. S. Lahman s.n.* (holotype: MO).
= Coryphanthafragrans Hester, Desert Pl. Life 13: 152. 1941. Type: United States, Texas, in a fertile, sandy loam valley, along the railroad right-of-way and Highway 90, a few miles west of Sanderson, 03 May 1940, *J. P. Hester s.n.* (holotype: DS [278622 image!]).
= Coryphantharosea Clokey, Madroño 7: 75. 1943. Coryphanthaviviparavar.rosea (Clokey) L.D.Benson, Cacti Ariz. ed. 3: 26. 1969. Escobariaviviparavar.rosea (Clokey) D.R.Hunt, Cact. Succ. J. Gr. Brit. 40: 13. 1978. Type: United States, Nevada, Clark County, between Kyle Canyon and Deer Creek. 24 Jun 1938, *I. W. Clokey 8038* (holotype: UC [905407 image!]; isotypes: F [52864 image!]; MEXU [86081 image!]; MICH [1127565 image!]; NY [120673 image!, 120672 image!], TEX [255617]).
= Coryphanthaoklahomensis Lahman, Cact. Succ. J. (Los Angeles) 21: 165. 1949. Escobariaoklahomensis (Lahman) Buxb., Oesterr. Bot. Z. 98: 78. 1951. Type: United States, Oklahoma, Caddo County, Range throughout western Oklahoma, *collector not mentioned* (lectotype, designated here Cact. Succ. J. (Los Angeles): 165. 1949: Illustration “fig. 107. Coryphanthaoklahomensis sp. nov. Photo by Jim Slack”).
= Coryphanthaalversoniivar.exaltissima Wiegand & Backeb., Cactaceae 5: 3001. 1961. Type: United States, California, ohne nähere Standortsangabe, *E. F. Wiegand s. n.* (lectotype, designated here Cactaceae (Backeberg): 3001. 1961: Illustration “Abb. 2817. Links: Coryphanthaalversonii (Coult.) Orc.; rechts: deren v. exaltissima Wieg & Backbg. (photo: E. F. Wiegand.)”).
= Coryphanthaviviparavar.kaibabensis P.C.Fisch., Cact. Succ. J. (Los Angeles) 51: 287. 1979. Escobariaviviparavar.kaibabensis (P.C.Fisch.) N.P.Taylor, Kakteen And. Sukk. 34: 139. 1983. Type. United States, Arizona, *P. C. Fischer 4094*. (holotype: UC).
= Coryphanthaviviparavar.buoflama P.C.Fisch., Cact. Succ. J. (Los Angeles) 52: 28. 1980. Escobariaviviparavar.buoflama (P.C.Fisch.) N.P.Taylor, Kakteen And. Sukk. 34: 140. 1983. Type. United States, Arizona, Yavapai County, 05 May 1979, *P. C. Fischer 6582*. (holotype: ARIZ; isotype: ASU [image 018464!]).
= Coryphanthaviviparavar.bisbeeanaf.sonorensis P.C.Fisch., Cact. Succ. J. (Los Angeles) 52: 191. 1980. Type. México, Sonora, 84 km north of Nacozari, on the road to U.S. border, 1430 m, 27 Apr 1971, *P. C. Fischer 4364*. (holotype: UC).

#### ﻿ Pelecyphora zilziana

Taxon classificationPlantaeCaryophyllalesCactaceae

(Boed.) D.Aquino & Dan.Sánchez comb. nov.

urn:lsid:ipni.org:names:77248965-1

≡ Coryphanthazilziana Boed., Monatsschr. Deutsch. Kakteen-Ges. 2: 233. 1930. Neobesseyazilziana (Boed.) Boed., Mammill.-Vergl.-Schluessel: 14. 1933. Neobesseyazilziana (Boed.) Boed. ex Backeb. & F.M.Knuth, Kaktus-ABC: 379. 1936. Escobariazilziana (Boed.) Backeb., Cactaceae 5: 2957. 1961. Type: Mexico, Coahuila, nördlich des Paila-Gebirges auf felsigen Hügeln von dunklem Eruptivgestein und auf Kalkhügeln sehr vereinzelt, 1928, *F. Ritter s.n.* (lectotype, designated here, Monatsschr. Deutsch. Kakteen-Ges.: 233. 1930b: Illustration “CoryphanthaZilziana Boed. sp. nov. natür. Grösse”).
= Escobariazilzianasubsp.fricii Halda & Sladk. in Acta Mus. Richnov., Sect. Nat. 7: 35. 2000. Type. México, Coahuila, Sierra de la Paila, in the vicinity of Castanos [Castaños], 2000 m, 13 Apr 1985, *J. J. Halda*, *J. Sladkovsky* 8504013520 (holotype: PR).

### ﻿*Coryphantha*

Phylogenetic analyses obtained here support the recognition of two subgenera in *Coryphantha* (clade C1 and clade C2), which are composed by two section (subclade A and subclade B) and five sections (subclades C to G), respectively. Also, 46 species and 12 subspecies of *Coryphantha*, are recognized. Asterisk (*) indicates species that were not included in the phylogenetic analyses. A taxonomic synthesis is presented.

#### ﻿ Coryphantha

Taxon classificationPlantaeCaryophyllalesCactaceae

(Engelm.) Lem., Cactées 32. 1868.

Mammillaria subgen. Coryphantha Engelm., Proc. Amer. Acad. Arts 3: 264. 1856.
Mammillaria subsect. Glanduliferae Salm-Dyck, Cact. Hort. Dyck. 1844: 13. 1845. Glandulifera (Salm-Dyck) Frič, Ceskoslov. Zahradn. Listy 1924: 122. 1924. nom. illeg.
Escobrittonia Doweld, Sukkulenty 3: 17. 2000. Type: Escobrittoniagracilis (L.Bremer & A.B.Lau) Doweld. Sukkulenty 3: 17. 2000.

##### Type.

*Coryphanthasulcata* (Engelm.) Britton & Rose

###### CoryphanthasubgenusCoryphantha

#### ﻿ Coryphantha section Corniferae

Taxon classificationPlantaeCaryophyllalesCactaceae

(Dicht & A.Lüthy) Dan.Sánchez & D.Aquino stat. nov.

urn:lsid:ipni.org:names:77248966-1

≡ Coryphanthaser.Corniferae Dicht & A.Lüthy, Coryphantha. Kakteen Nordamer. 91. 2003. ≡ Coryphanthasubser.Corniferae Dicht & A.Lüthy, Cactaceae Syst. Init. 11: 19. 2001. Type: Coryphanthacornifera (DC.) Lem., Cactées 35. 1868.

#### ﻿ Coryphantha section Gracilicoryphantha

Taxon classificationPlantaeCaryophyllalesCactaceae

Dicht & A.Lüthy, Cactaceae Syst. Init. 11: 21, 2001.

##### Type.

*Coryphanthagracilis* Bremer & A.B.Lau, Cact. Succ. J. (Los Angeles) 49: 72. 1977.

#### ﻿ Coryphantha subser. Delaetianae

Taxon classificationPlantaeCaryophyllalesCactaceae

Dicht & A.Lüthy, Cactaceae Syst. Init. 11: 20. 2001.

##### Type.

*Coryphanthadelaetiana* (Quehl) A.Berger, Kakteen: 270, 339. 1929.

#### ﻿ Coryphantha subser. Neglectae

Taxon classificationPlantaeCaryophyllalesCactaceae

Dicht & A.Lüthy, Cactaceae Syst. Init. 11: 20. 2001.

##### Type.

*Coryphanthaneglecta* L.Bremer, Cact. Suc. Mex. 24: 3. 1979.

##### Species included

(*inserta sedis). *Coryphanthacompacta* (Engelm.) Orcutt, *C.cornifera* (DC.) Lem., *C.delaetiana* (Quehl) A.Berger, *C.delicata* L.Bremer, *C.gracilis L. Bremer & A.B.Lau, *C.hintoniorum* Dicht & A.Lüthy, C.hintoniorumsubsp.geofreyii Dicht & A.Lüthy, *C.maiz-tablasensis* Backeb., *C.neglecta* L.Bremer, *C.nickelsiae* (K.Brandegee) Britton & Rose, *C.pseudoechinus* Boed., *C.pseudonickelsiae* Backeb., *C.pulleineana (Backeb.) Glass, *C.ramillosa* Cutak, C.ramillosasubsp.santarosa Dicht & A.Lüthy *C.recurvata* (Engelm.) Britton & Rose and C.recurvatasubsp.canatlanensis Dicht & A.Lüthy.

#### ﻿ Coryphantha section Coryphantha

Taxon classificationPlantaeCaryophyllalesCactaceae

Coryphantha ser. Salinenses Dicht & A.Lüthy, Cactaceae Syst. Init. 11: 15. 2001. Type: Coryphanthasalinensis (Poselg.) Dicht & A.Lüthy, Kakteen And. Sukk. 49: 257.

##### Type.

*Coryphanthasulcata* (Engelm.) Britton & Rose, Cactaceae 4: 48. 1923.

##### Species included.

*Coryphanthadifficilis* (Quehl) Orcutt, *C.echinus* (Engelm.) Britton & Rose, *C.kracikii* Halda, Chalupa & Kupčák, *C.salinensis* (Poselg.) Dicht & A.Lüthy, *C.sulcata* (Engelm.) Britton & Rose, and *C.werdermannii* Boed.

#### ﻿ Coryphantha section Durangenses

Taxon classificationPlantaeCaryophyllalesCactaceae

Dan.Sánchez & D.Aquino sect. nov.

urn:lsid:ipni.org:names:77248967-1

##### Type.

*Coryphanthadurangensis* Britton & Rose, Cactaceae (Britton & Rose) 4: 42. 1923.

##### Species included.

*Coryphanthadurangensis* (Runge ex K.Schum.) Britton & Rose, C.durangensissubsp.cuencamensis (L.Bremer) Dicht & A.Lüthy, and *C.longicornis* Boed.

#### ﻿ Coryphantha section Pycnacanthae

Taxon classificationPlantaeCaryophyllalesCactaceae

(Dicht & A.Lüthy) Dan.Sánchez & D.Aquino stat. nov.

urn:lsid:ipni.org:names:77248968-1

Coryphantha ser. Retusae Dicht & A.Lüthy, Cactaceae Syst. Init. 11: 14. 2001.Type: Coryphantharetusa (Pfeiff.) Britton & Rose, Cactaceae 4: 38. 1923.

##### Basionym.

Coryphanthaser.Pycnacanthae Dicht & A.Lüthy, Cactaceae Syst. Init. 11: 15. 2001.

##### Type.

*Coryphanthapycnacantha* (Mart.) Lem., Cactées: 35. 1868.

##### Species included

(*inserta sedis): *Coryphanthabumamma* (C.Ehrenb.) Britton & Rose, *C.calipensis* Bravo ex S.Arias, U.Guzmán & S.Gama, *C.elephantidens* (Lem.) Lem., *C.greenwoodii* Bravo, *C.pallida* Britton & Rose, *C.pseudoradians Bravo, *C.pycnacantha* (Mart.) Lem., *C.retusa* (Pfeiff.) Britton & Rose, and *C.tripugionacantha* A.B. Lau.

#### ﻿ Coryphantha section Robustispina

Taxon classificationPlantaeCaryophyllalesCactaceae

Dicht & A.Lüthy, Cactaceae Syst. Init. 11: 9. 2001.

##### Type.

*Coryphantharobustispina* (Ant.Schott ex Engelm.) Britton & Rose, Cactaceae 4: 33. 1923.

##### Species included.

*Coryphantharobustispina* (Ant.Schott ex Engelm.) Britton & Rose, C.robustispinasubsp.scheeri (Lem.) N.P. Taylor, and *C.poselgeriana* (A.Dietr.) Britton & Rose.

#### ﻿ Coryphantha subgenus Neocoryphantha

Taxon classificationPlantaeCaryophyllalesCactaceae

Backeb. ex Dicht & A. Lüthy, Cactaceae Syst. Init. 11: 8, 2001.

##### Type.

*Coryphanthaclavata* (Scheidw.) Backeb., Jahrb. Deutsch. Kakt. Ges. 1941: 61. 1942.

#### ﻿ Coryphantha section Clavatae

Taxon classificationPlantaeCaryophyllalesCactaceae

(Dicht & A. Lüthy) Dan.Sánchez & D.Aquino stat. nov.

urn:lsid:ipni.org:names:77248969-1

≡ CoryphanthaSer.Clavatae Dicht & A.Lüthy, Cactaceae Syst. Init. 11: 11. 2001. Type: Coryphanthaclavata (Scheidw.) Backeb., Jahrb. Deutsch. Kakt. Ges. 1941: 61. 1942.
Coryphantha sect. Ottonis Dicht & A.Lüthy, Cactaceae Syst. Init. 11: 13. 2001. Type: Coryphanthaottonis (Pfeiff.) Lem., Cactées 34. 1868.

##### Species included.

*Coryphanthaclavata* (Scheidw.) Backeb., C.clavatasubsp.stipitata (Scheidw.) Dicht & A.Lüthy, *C.erecta* (Lem.) Lem., *C.georgii* Boed., *C.glassii* Dicht & A.Lüthy, *C.jalpanensis* Buchenau, *C.octacantha* (DC.) Britton & Rose, *C.ottonis* (Pfeiff.) Lem., *C.potosiana* (Jacobi) Glass & R.A.Foster, and *C.vogterriana* Werderm. & Boed.

#### ﻿ Coryphantha section Echinoideae

Taxon classificationPlantaeCaryophyllalesCactaceae

(Dicht & A. Lüthy) Dan.Sánchez & D.Aquino stat. nov.

urn:lsid:ipni.org:names:77248971-1

≡ CoryphanthaSer.Echinoideae Dicht & A.Lüthy, Cactaceae Syst. Init. 11: 10. 2001. Type: Coryphanthaechinoidea Britton & Rose, Cactaceae (Britton & Rose) 4: 30. 1923.

##### Species included.

*Coryphanthaechinoidea* (Quehl) Britton & Rose, *C.glanduligera* (Otto & A.Dietr.) Lem., *C.vaupeliana* Boed., and *C.wolhschlageri* Holzeis.

### ﻿New neotypes and lectotypes

Furthermore, two neotypes and three lectotypes are proposed. For a more extensive review of the accepted names in *Coryphantha*, see Dicht and Lüthy (2005).

#### ﻿ Coryphantha potosiana

Taxon classificationPlantaeCaryophyllalesCactaceae

(Jacobi) Glass & R.A.Foster, Cact. Succ. J. (Los Angeles) 43: 7. 1971.

≡ Mammillariapotosiana Jacobi, Allg. Gartenzeitung (Otto & Dietrich) 24: 92. 1856. Coryphanthapotosiana (Jacobi) Glass & R.A.Foster ex Rowley, Rep. Pl. Succ. 21: 8. 1972. Type: México, San Luís Potosí, 1847, *Jacobi & Galeottii s.n.* (not preserved). Neotype designated here: México, San Luís Potosí, Villa de Arriaga, Rincón de Silva, 2200 m, 23 Jun 1983, *R. Hernández s.n.* (MEXU: 363520!).

#### ﻿ Coryphantha ottonis

Taxon classificationPlantaeCaryophyllalesCactaceae

(Pfeiff.) Lem., Cactées: 34. 1868.

≡ Mammillariaottonis Pfeiff., Allg. Gartenzeitung 6: 274. 1838. Cactusottonis (Pfeiff.) Kuntze, Revis. Gen. Pl. 1: 261. 1891. Type: Not designed. Neotype designated here: México, Estado Mex., Polotitlán, Colonia Doctores, a unos 2 km al E de la Carretera de Cuota México Querétaro, a la altura del km 130, 2000 m, 27 May 1977, *H. Sánchez-Mejorada 2728* (MEXU: 204376!).
= Mammillariaasterias Cels ex Salm-Dyck, Cact. Hort. Dyck.: 129. 1850. Coryphanthaasterias (Cels) Hübner, Kakteenfreund (Beil) 2: 8. 1933. Type: Not designated.
= Echinocactusottonianus Poselg., Allg. Gartenzeitung 21: 102. 1853. Coryphanthaottonianus (Poselg.) Y.Itô, Cactaceae: 553. 1981. Type: Not designated.
= Mammillariabussleri Mundt ex K.Schum., Monatsschr. Kakteenk. 12: 47. 1902. Coryphanthabussleri (Mundt) Scheinvar, Phytologia 49: 313. 1981. Type: México, *Anonymous s.n*. (lectotype, designated here, Monatsschr. Kakteenk.: 47. 1902: Illustration “Mamillatia Bussleri Mundt. Nach einer von dem Herrn Autor angefertigten Photographie”).
= Mammillariagolziana F.Haage ex R.E.Kunze, Monatsschr. Kakteenk. 19: 100. 1909. Type: México, Zacatecas, *Anonymous s.n*. (lectotype, designated here, Monatsschr. Kakteenk.: 100. 1909: Illustration “Mamillatia Golziana” Ferd. Haage jun. Nach einer von Herrn Dr R. E. Kunze in Phoenix (Arizona) aufgenommenen Photographie”).
= Mammillariaguerkeana Boed., Monatsschr. Kakteenk. 24: 52. 1914. Coryphanthaguerkeana (Boed.) Britton & Rose, Cactaceae 4: 29. 1923. Type: México, Durango, 1911, *F. De Laet s.n*. (lectotype, designated here: US [2975102 image!]).

## Supplementary Material

XML Treatment for Cochemiea mazatlanensis

XML Treatment for Pelecyphora

XML Treatment for Pelecyphora abdita

XML Treatment for Pelecyphora abdita subsp. tenuispina

XML Treatment for Pelecyphora alversonii

XML Treatment for Pelecyphora aselliformis

XML Treatment for Pelecyphora chihuahuensis

XML Treatment for Pelecyphora chihuahuensis subsp. henricksonii

XML Treatment for Pelecyphora cubensis

XML Treatment for Pelecyphora dasyacantha

XML Treatment for Pelecyphora dasyacantha subsp. chaffeyi

XML Treatment for Pelecyphora duncanii

XML Treatment for Pelecyphora emskoetteriana

XML Treatment for Pelecyphora hesteri

XML Treatment for Pelecyphora hesteri subsp. grata

XML Treatment for Pelecyphora laredoi

XML Treatment for Pelecyphora lloydii

XML Treatment for Pelecyphora macromeris

XML Treatment for Pelecyphora macromeris subsp. runyonii

XML Treatment for Pelecyphora minima

XML Treatment for Pelecyphora missouriensis

XML Treatment for Pelecyphora missouriensis subsp. asperispina

XML Treatment for Pelecyphora robbinsorum

XML Treatment for Pelecyphora sneedii

XML Treatment for Pelecyphora sneedii subsp. orcuttii

XML Treatment for Pelecyphora strobiliformis

XML Treatment for Pelecyphora tuberculosa

XML Treatment for Pelecyphora vivipara

XML Treatment for Pelecyphora zilziana

XML Treatment for Coryphantha

XML Treatment for Coryphantha section Corniferae

XML Treatment for Coryphantha section Gracilicoryphantha

XML Treatment for Coryphantha subser. Delaetianae

XML Treatment for Coryphantha subser. Neglectae

XML Treatment for Coryphantha section Coryphantha

XML Treatment for Coryphantha section Durangenses

XML Treatment for Coryphantha section Pycnacanthae

XML Treatment for Coryphantha section Robustispina

XML Treatment for Coryphantha subgenus Neocoryphantha

XML Treatment for Coryphantha section Clavatae

XML Treatment for Coryphantha section Echinoideae

XML Treatment for Coryphantha potosiana

XML Treatment for Coryphantha ottonis

## Figures and Tables

**Figure A1.**
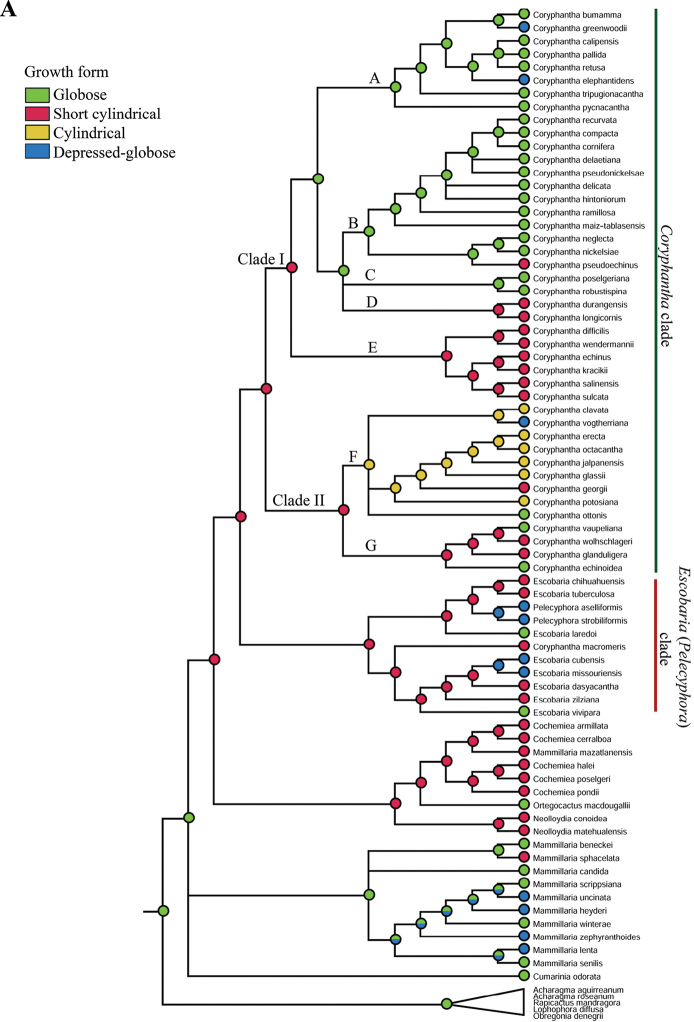
Ancestral states reconstruction in Coryphantha and related genera **A** growth form **B** groove on the tubercle in mature plant **C** extrafloral glands at or near the axil **D** position of the flowers **E** margin of the outer tepals **F** color of the mature fruit **G** type of cortex **H** multicellular sculpture of the lateral side of the seed.

**Figure A1.**
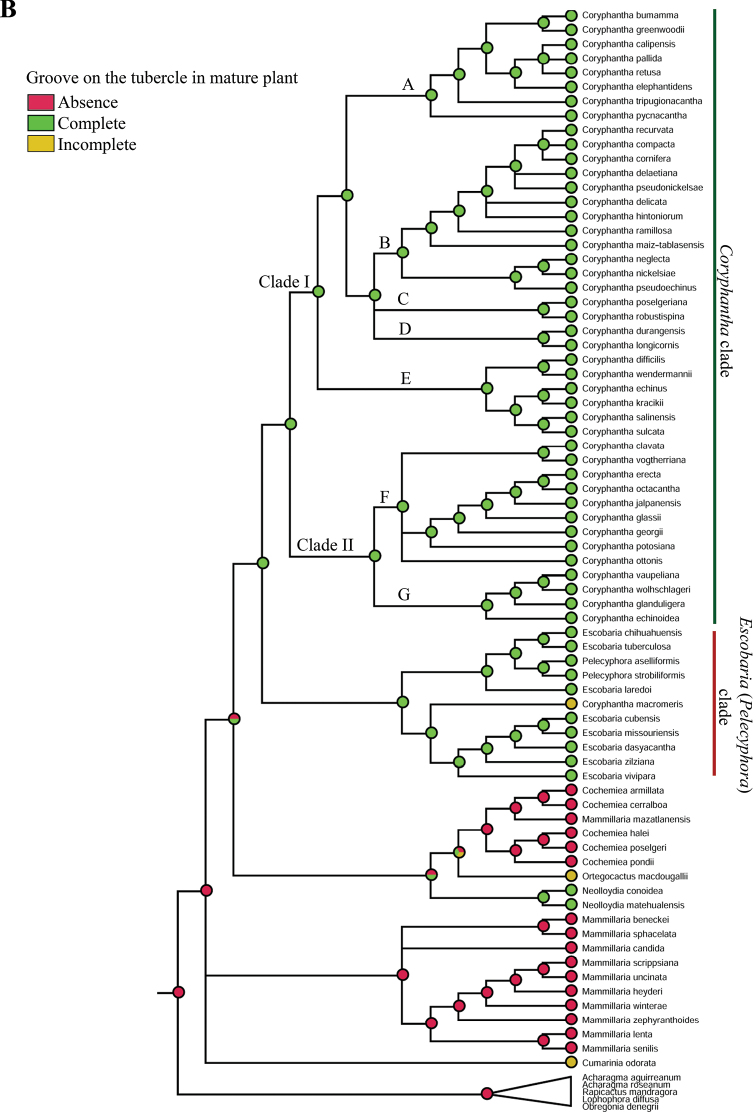
Continued.

**Figure A1.**
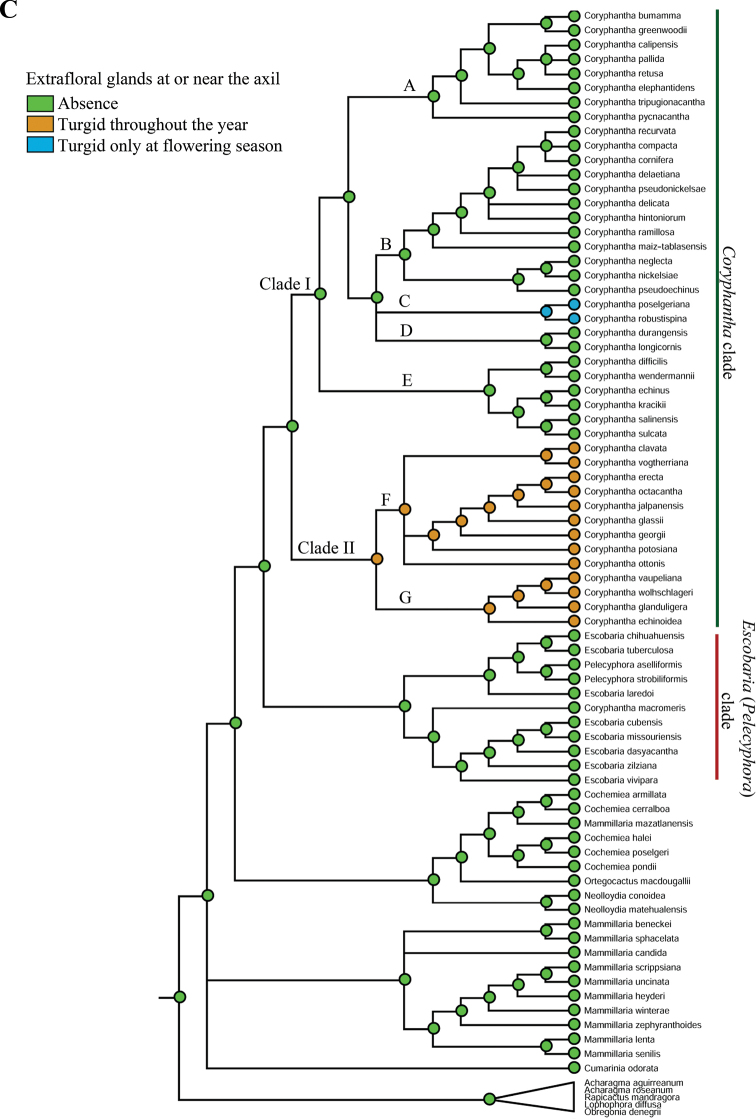
Continued.

**Figure A1.**
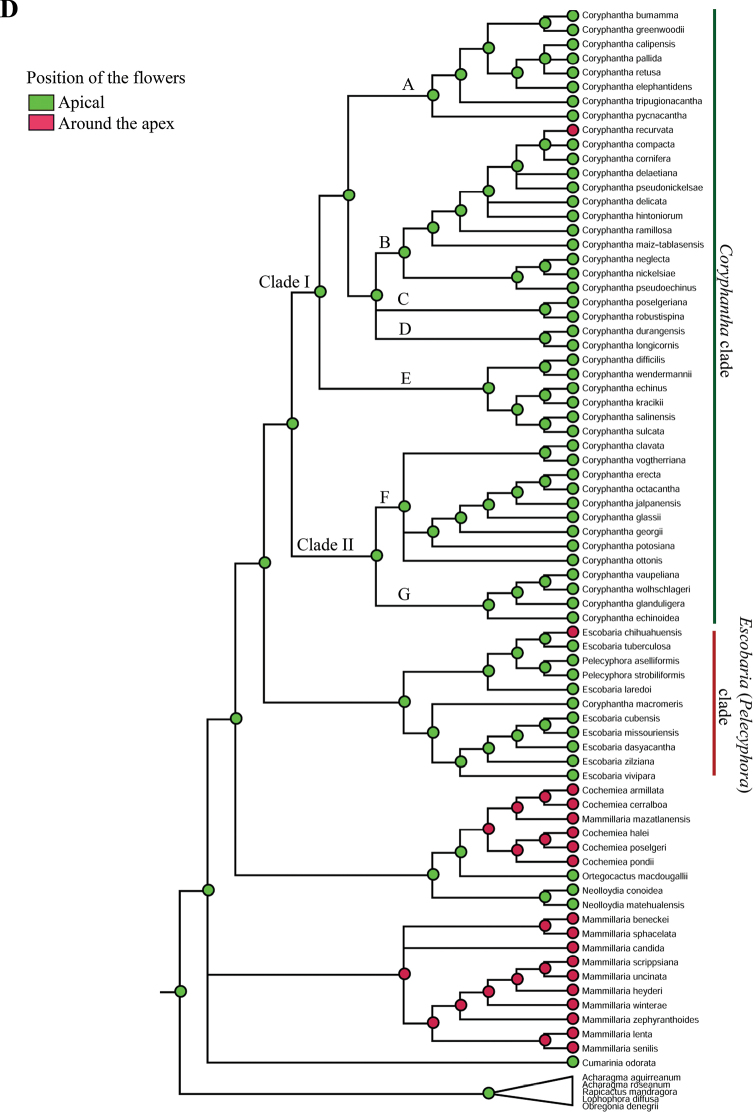
Continued.

**Figure A1.**
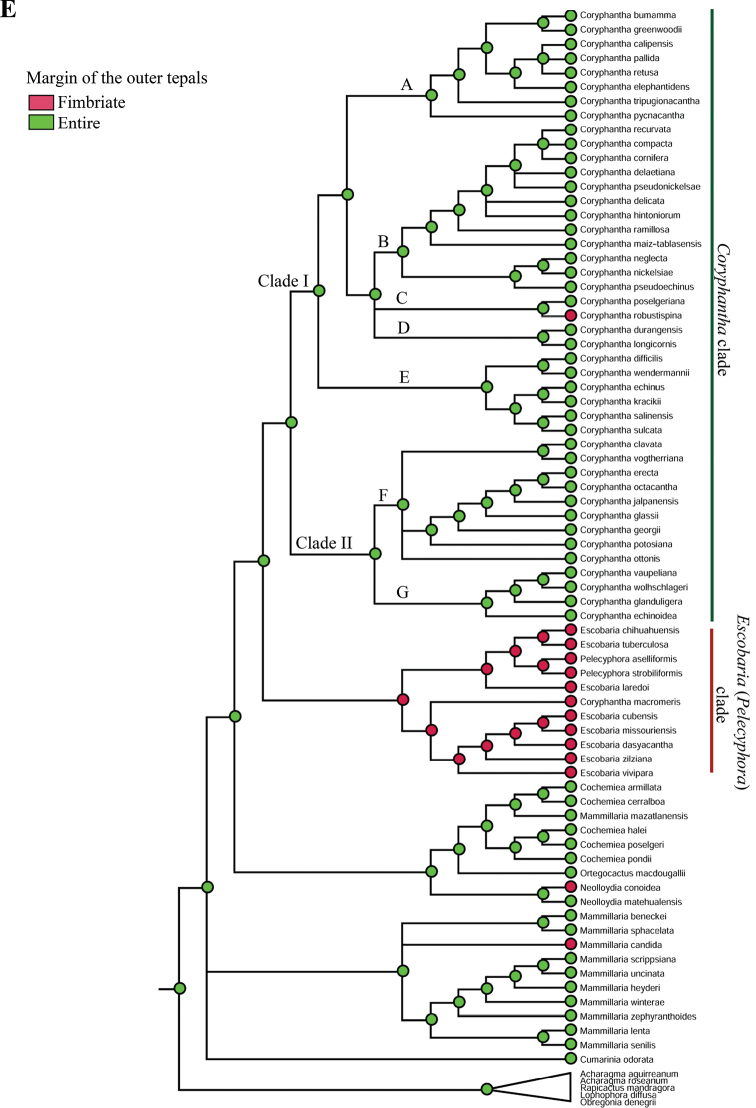
Continued.

**Figure A1.**
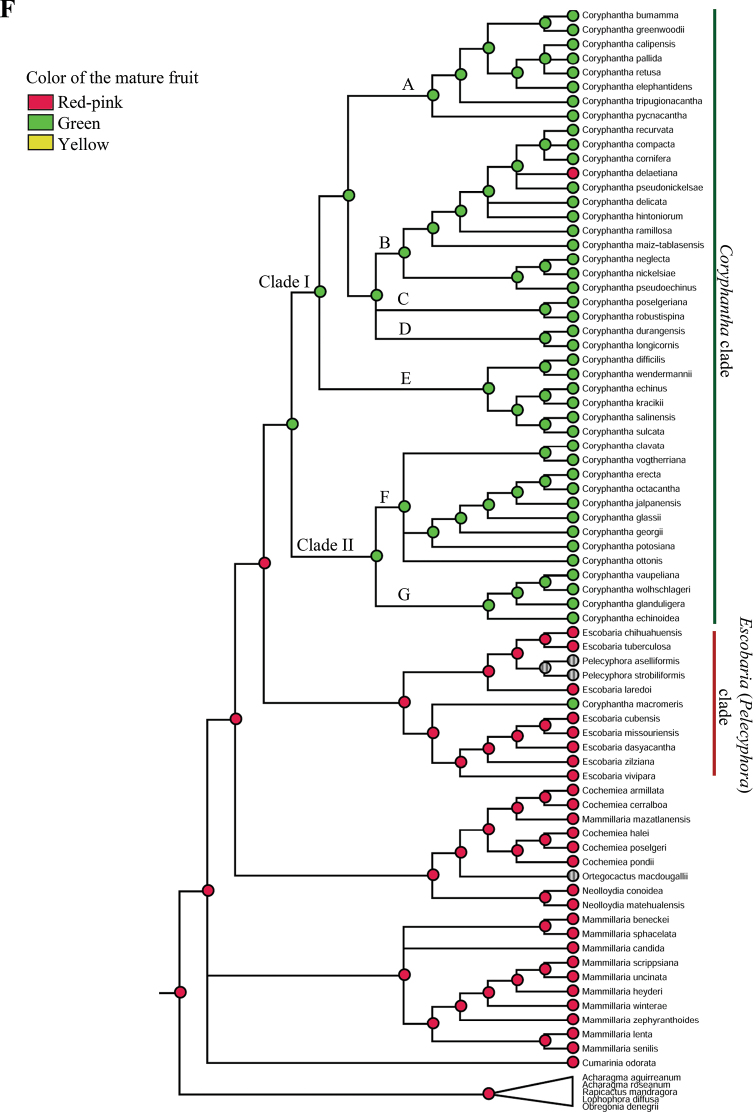
Continued.

**Figure A1.**
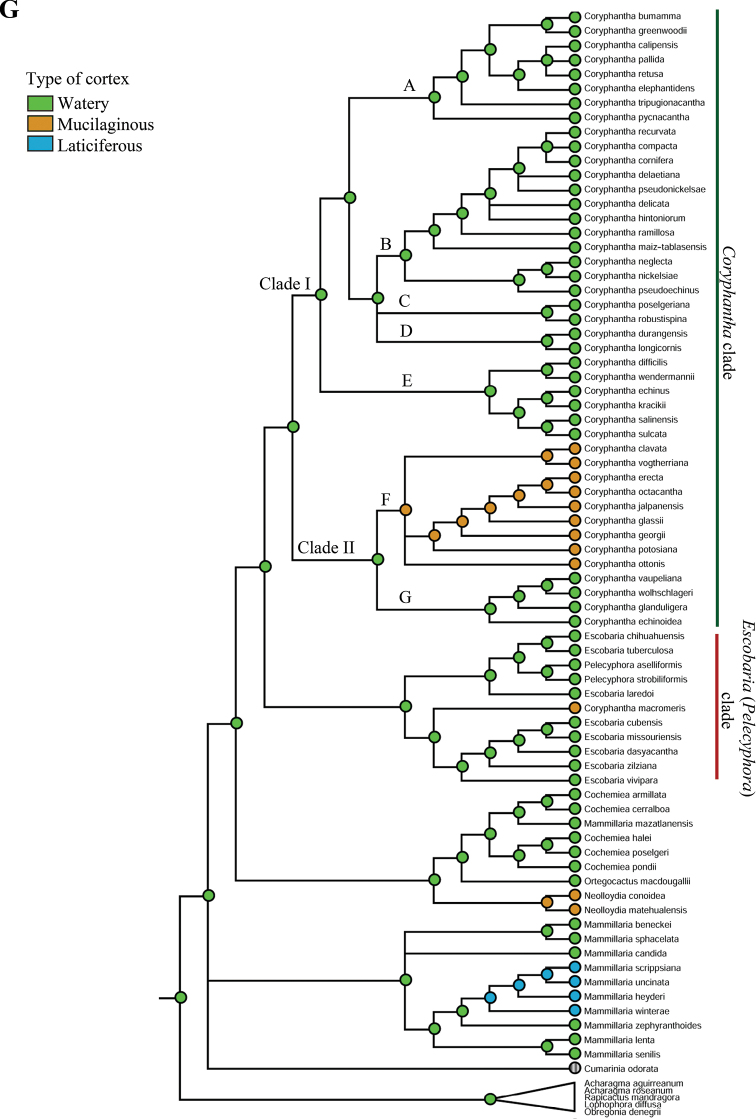
Continued.

**Figure A1.**
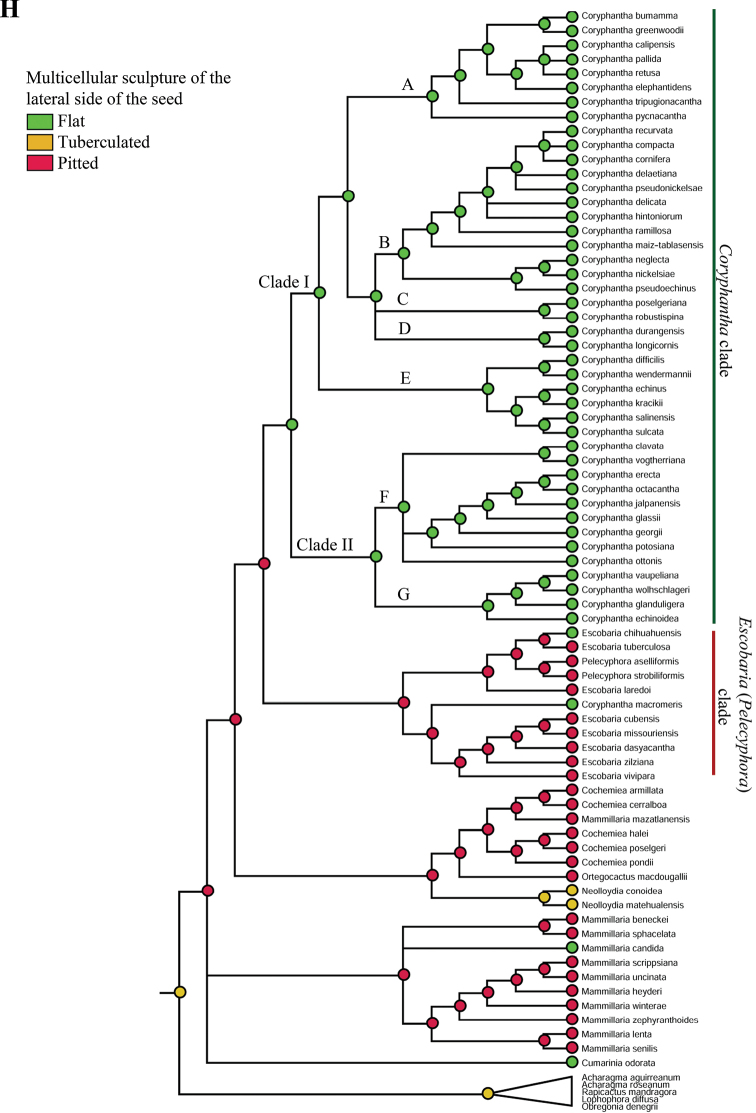
Continued.

## ﻿Appendix 1

Accessions included in this study, presented in alphabetical order, and following this format: taxon name in bold, country, estate, collector, collecting number (HERBARIUM ACRONYM) and Gen Bank accession as follow: *matK*/ *rbcL*/ *psbA-trnH*/ *rpl16*/ *trnL-F*. A dash (–) indicates that the locus was not sequenced for that specimen. Living voucher specimens are identified by their specimen number in cultivation at Desert Botanical Garden (DES), Jardín Botánico, Instituto de Biología, Universidad Nacional Autónoma de México (JB, UNAM), and El Charco del Ingenio, A.C. ND: no data.

***Acharagmaaguirreanum*** (Glass & R.A.Foster) Glass. Mexico, Coahuila, *S. Arias 1459* (MEXU): MK449027/ MK449085/ MK449274/ AF267915/ MK449212; ***Acharagmaroseanum*** (Boed.) E.F.Anderson. Mexico, Coahuila, *C. Glass 6443* (MEXU): MK449028/ MK449086/ MK449275/ MK449151/ MK449213; ***Cochemieaarmillata*** (K.Brandegee) P.B.Breslin & Majure, cult.: FN997315/ –/ AY545949/AY545240/–; ***Cochemieacerralboa*** (Britton & Rose) P.B.Breslin & Majure, cult.: FN997003/ –/ AY545364/ –/ –; ***Cochemieahalei*** (K.Brandegee) Walton, Mexico, *S. Arias 1287* (MEXU): OL513239/ OL513243/ OL513236/ OL513246/ –; ***Cochemieapondii*** (Greene) Walton, Mexico, *S. Arias 1862* (MEXU): OL513240/ OL513244/ OL513237/ OL513247/ –, V. Alvarado s.n.: –/ –/ –/ –/ HM041244; ***Cochemieaposelgeri*** (Hildm.) Britton & Rose, Mexico, Baja California, *S. Arias 1824* (MEXU): OL513241/ OL513245/ OL513238/ OL513248/ -, T. Hernández p106: –/ –/ –/ –/ HM041245; ***Coryphanthabumamma*** Britton & Rose. Mexico, Guerrero, *B. Vázquez 2628* (MEXU): OK340224/ OK340287/ OK340349/ OK340410/ OK340462; ***Coryphanthacalipensis*** Bravo ex S.Arias, Gama & U.Guzman. Mexico, Oaxaca, *B. Vázquez 2555* (MEXU): OK340225/ OK340288/ OK340350/ OK340411/ OK340463; ***Coryphanthaclavata*** (Scheidw.) Backeb. Mexico, San Luis Potosí, *T. Terrazas 886* (MEXU): OK340227/ OK340290/ OK340352/ –/ OK340465; ***Coryphanthacompacta*** (Engelm.) Orcutt. Mexico, Chihuahua, *B. Vázquez 2608* (MEXU): OK340228/ OK340291/ OK340353/ OK340413/ OK340466; ***Coryphanthacornifera*** Lem. Mexico, Querétaro, *S. Arias 1700* (MEXU): OK340229/ OK340292/ OK340354/ OK340414/ OK340467; ***Coryphanthadelaetiana*** A.Berger. Mexico, Durango, *S. Arias 1901* (MEXU): OK340231/ OK340294/ OK340356/ OK340416/ –; ***Coryphanthadelicata*** L.Bremer. Mexico, San Luis Potosí, *B. Vázquez 2546* (MEXU), OK340232/ OK340295/ OK340357/ OK340417/ OK340469; ***Coryphanthadifficilis*** Orcutt. Mexico, Coahuila, *B. Vázquez 2541* (MEXU): OK340233/ OK340296/ OK340358/ –/ OK340470; ***Coryphanthadurangensis*** Britton & Rose. Mexico, Durango, *B. Vázquez 2626* (MEXU): OK340234/ OK340297/ OK340359/ OK340418/ OK340471; ***Coryphanthadurangensis subsp. cuencamensis*** (L.Bremer) Dicht & A.Lüthy. Mexico, Durango, *B. Vázquez 2627* (MEXU): OK340230/ OK340293/ OK340355/ OK340415/ OK340468; ***Coryphanthaechinoidea*** Britton & Rose. Mexico, San Luis Potosí, *B. Vázquez 2514* (MEXU): OK340235/ OK340298/ OK340360/ –/ OK340472; ***Coryphanthaechinus*** (Engelm.) Orcutt. Mexico, Chihuahua, *S. Arias 2072* (MEXU): OK340236/ OK340299/ OK340361/ OK340419/ OK340473; ***Coryphanthaelephantidens*** Lem. Mexico, Guerrero, *B. Vázquez 2629* (MEXU): OK340237/ OK340300/ OK340362/ OK340420/ OK340474; ***Coryphanthaerecta*** Lem. Mexico, Querétaro, *S. Arias 1684* (MEXU): OK340238/ OK340301/ OK340363/ OK340421/ OK340475; ***Coryphanthageorgii*** Boed. Mexico, San Luis Potosí, *B. Vázquez 2517* (MEXU): OK340239/ OK340302/ OK340364/ OK340422/ OK340476; ***Coryphanthaglanduligera*** (Otto & A.Dietr.) Lem. Mexico, San Luis Potosí, *B. Vázquez 2547* (MEXU): OK340240/ OK340303/ OK340365/ OK340423/ OK340477; ***Coryphanthaglassii*** Dicht & A.Lüthy. Mexico, San Luis Potosí, *B. Vázquez 2525* (MEXU): OK340241 / OK340304/ OK340366/ OK340424/ OK340478; ***Coryphanthagreenwoodii*** Bravo. Mexico, Veracruz, *B. Vázquez 2630* (MEXU): OK340242/ OK340305/ OK340367/ OK340425/ OK340479; ***Coryphanthahintoniorum*** Dicht & A.Lüthy. Mexico, Nuevo León, *B. Vázquez 2539* (MEXU): OK340243/ OK340306/ OK340368/ OK340426/ OK340480; ***Coryphanthajalpanensis*** Franc.G.Buchenau. Mexico, Querétaro, *B. Vázquez 2586* (MEXU): OK340244/ OK340307/ OK340369/ OK340420/ OK340481; ***Coryphanthakracikii*** Halda, Chalupa & Kupčák. Mexico, Durango, *B. Vázquez 2618* (MEXU): OK340245/ OK340308/ OK340370/ –/ OK340482; ***Coryphanthalongicornis*** Boed. Mexico, Durango, *B. Vázquez 2623* (MEXU): OK340246/ OK340309/ OK340371/ OK340428/ OK340483; ***Coryphanthamacromeris*** (Engelm.) Lem. Mexico, San Luis Potosí, B. *Goettsch 169* (MEXU): FN997086 / –/ –/ –/ –. Mexico, Chihuahua, *S. Arias 1788* (MEXU): OK340247/ OK340310/ OK340372/ OK340429/ –; ***Coryphanthamaiz-tablasensis*** Fritz Schwarz. JE280502 (cult. JB, UNAM), ND: OK340248/ OK340311/ OK340373/ OK340430/ OK340484; ***Coryphanthaneglecta*** L.Bremer. Mexico, Coahuila, *S. Arias 2116* (MEXU): OK340249/ OK340312/ OK340374/ OK340431/ OK340485; ***Coryphanthanickelsiae*** (K.Brandegee) Britton & Rose. Mexico, Nuevo León, *B. Vázquez 2565* (MEXU): OK340250/ OK340313/ OK340375/ OK340432/ OK340486; ***Coryphanthaoctacantha*** Britton & Rose. Mexico, Hidalgo, *B. Vázquez 2531* (MEXU): OK340251/ OK340314/ OK340376/ OK340433/ OK340487; ***Coryphanthaottonis*** Lem. Mexico, Estado de México, *B. Vázquez 2588* (MEXU): OK340252/ OK340315/ OK340377/ –/ OK340488; ***Coryphanthapallida*** Britton & Rose. Mexico, Puebla, *B. Vázquez 2552* (MEXU): OK340253/ OK340316/ OK340378/ OK340434/ OK340489; ***Coryphanthaposelgeriana*** Britton & Rose. Mexico, Coahuila, B. *Vázquez 2544* (MEXU): OK340254/ OK340317/ OK340379/ OK340435/ OK340490; ***Coryphanthapotosiana*** (Jac.) Glass & R.A.Foster ex G.D.Rowley. Mexico, San Luis Potosí, *U. Guzmán 2771* (MEXU): OK340255/ OK340318/ OK340380/ OK340436/ OK340491; ***Coryphanthapseudoechinus*** Boed. Mexico, Coahuila, *B. Vázquez 2542* (MEXU): OK340256/ OK340319/ OK340381/ OK340438/ OK340492; ***Coryphanthapseudonickelsiae*** Backeb. Mexico, Durango, *B. Vázquez 2620* (MEXU): OK340257/ OK340320/ OK340382/ OK340438/ OK340493; ***Coryphanthapycnacantha*** (Mart.) Lem. Mexico, Estado de México, *B. Vázquez 2589* (MEXU): OK340258/ OK340321/ OK340383/ OK340439/ OK340494; ***Coryphantharamillosa*** Cutak. Mexico, Chihuahua, *S. Arias 2070* (MEXU): OK340259/ OK340322/ OK340384/ –/ OK340495; ***Coryphantharecurvata subsp.canatlanensis*** Dicht & A.Lüthy. Mexico, Durango, *S. Arias 1893* (MEXU): OK340226/ OK340289/ OK340351/ OK340412/ OK340464; ***Coryphantharetusa*** Britton & Rose. Mexico, Oaxaca, *B. Vázquez 2558* (MEXU): OK340260/ OK340323/ OK340385/ OK340440/ OK340496; ***Coryphantharobustispina*** (A.Schott ex Engelm.) Britton & Rose. Mexico, Chihuahua, *B. Vázquez 2581* (MEXU): OK340261/ OK340324/ OK340386/ OK340441/ OK340497; ***Coryphanthasalinensis*** (Poselg.) Dicht & A.Lüthy. Mexico, Nuevo León, *B. Vázquez 2566* (MEXU): OK340262/ OK340325/ OK340387/ –/ OK340498; ***Coryphanthasulcata*** (Engelm.) Britton & Rose. Mexico, Nuevo León, *S. Arias 2162* (MEXU): OK340263/ OK340326/ OK340388/ OK340442/ OK34049; ***Coryphanthatripugionacantha*** A.B.Lau. cult. (JB El Charco del Ingenio, AC), ND: FN997162/ –/ –/ –/ –; ***Coryphanthavaupeliana*** Boed. Mexico, Tamaulipas, *B. Vázquez 2564* (MEXU): OK340264/ OK340327/ OK340389/ –/ OK340500; ***Coryphanthavogtherriana*** Werderm. & Boed. Mexico, San Luis Potosí, *B. Vázquez 2538* (MEXU): OK340265/ OK340328/ OK340390/ OK340443/ OK340501; ***Coryphanthawedermannii*** Boed. Mexico, Coahuila, *S. Arias 2104* (MEXU): OK340266/ OK340329/ OK340391/ –/ OK340502; ***Coryphanthawohlschlageri*** Holzeis. Mexico, San Luis Potosí, *B. Vázquez 2587* (MEXU): OK340267/ OK340330/ OK340392/ OK340444/ OK340503; ***Cumariniaodorata*** (Boed.) Buxb. Mexico, San Luis Potosí, *J. Reyes 5940* (cult. JB, UNAM): MK449037/ MK449094/ MK449284/ MK449160/ MK449222; ***Echinocactusplatyacanthus*** Link & Otto. Mexico, Querétaro, *S. Arias 1679* (MEXU): OK340223/ OK340286/ –/ OK340409/ –; ***Epithelanthaspinosior*** C. Schmoll. Mexico, Coahuila, *S. Arias 1507* (MEXU): MK449039/ MK449096/ MK449286/ MK449162/ MK449224; ***Escobariachihuahuensis*** Britton & Rose. Mexico, Chihuahua, *S. Arias 1908* (MEXU): OK340271/ OK340334/ OK340395/ OK340448/ OK340506; ***Escobariacubensis*** (Britton & Rose) D.R.Hunt. Cuba, Holguin, D. Barios 24 (HAJB): OL513242/ – / MK284092/ OL513249/ MK284152; ***Escobariadasyacantha*** (Engelm.) Britton & Rose. Mexico, Coahuila, *S. Arias 1955* (MEXU): OK340272/ OK340335/ OK340396/ OK340449/ OK340507; ***Escobarialaredoi*** (Glass & R.A.Foster) N.P.Taylor. Mexico, Coahuila, *S. Arias 1951* (MEXU): MK449040/ MK449097/ MK449287/ MK449163/ MK449225; ***Escobariamissouriensis*** (Sweet) D.R.Hunt. Mexico, Nuevo León, *S. Arias 1945* (MEXU): MK449041/ MK449098/ MK449288/ MK449164/ MK449226; ***Escobariatuberculosa*** (Engelm.) Britton & Rose. cult. (JB El Charco del Ingenio, AC), ND: FN997185/ –/ –/ –/ –. cult. DES 1986-0619-0101 (ISC), ND: –/ –/ AY545343/ AY545235/ –; ***Escobariavivipara*** (Nutt.) Buxb. United States, Nevada, *Andrew Salywon 1885* (DES): –/ –/ KC196847/ KC196809/ –. CCDB-23325-H02 (CANA), Canada, Saskatchewan: –/ MG246257/ –/ –/ –. *McElroy* s.n. (MEXU), ND: FN997563/ –/ –/ –/ –; ***Escobariazilziana*** (Boed.) Backeb. cult. s.n. (JB El Charco del Ingenio, AC), ND: –/ –/ AY545344/ AY545236/ –; ***Escobariazilziana*** (Boed.) Backeb. cult. s.n. (JB El Charco del Ingenio, AC), ND: FN997193/ –/ –/ –/ –; ***Ferocactusalamosanus*** (Britton & Rose) Britton & Rose. Mexico, Sonora, *S. Arias 1846* (MEXU): OK340273/ OK340336/ OK340397/ OK340450/ OK340508; ***Ferocactusglaucescens*** Britton & Rose. Mexico, Querétaro, *S. Arias 1701* (MEXU): OK340274/ OK340337/ OK340398/ OK340451/ OK340509; ***Ferocactusrecurvus*** (Mill.) Borg. Mexico, Puebla, *S. Arias 1794* (MEXU): OK340275/ OK340338/ OK340399/ OK340452/ OK340510; ***Kadenicarpushorripilus*** (Lem.) Vázquez-Sánchez. Mexico, Hidalgo, *J.M Chalet 204* (cult. JB, UNAM): MK449042/ MK449121/ MK449311/ MK449185/ MK449247; ***Lophophoradiffusa*** (Croizat) Bravo. Mexico, Querétaro, *S. Arias 35* (MEXU): MK449046/ MK449100/ MK449290/ MK449166/ MK449228; ***Mammillariabeneckei*** Ehrenb, cult.: FN997206/ –/ AY545353/AF267944/ AJ583216; ***Mammillariaheyderi*** Muehlenpf. Mexico, San Luis Potosí, *T. Terrazas 829* (MEXU): OK340276/ OK340339/ OK340400/ OK340453/ OK340511; ***Mammillarialenta*** K.Brandegee. Mexico, Coahuila, MX *T. Terrazas 907* (MEXU): MK449047/ MK449102/ MK449292/ MK449167/ MK449230; ***Mammillariamazatlanensis*** K.Schum.: FN997141/ –/ AY545407/ AY545287/ AJ583226; ***Mammillariascrippsiana*** (Britton & Rose) Orcutt. Mexico, Nayarit, *S. Arias 1886* (MEXU): OK340277/ OK340340/ OK340401/ OK340454/ OK340512; ***Mammillariasenilis*** Lodd. ex Salm-Dyck. Mexico, Durango, *S. Arias 1890* (MEXU): OK340278/ OK340341/ OK340402/ OK340455/ OK340513; ***Mammillariasphacelata*** Mart.: FN997483/ –/ AY545442/ AY545320/ –; ***Mammillariauncinata*** Zucc. ex Pfeiff. Mexico, Guanajuato, *S. Arias 1687* (MEXU): OK340279/ OK340342/ OK340403/ OK340456/ OK340514; ***Mammillariawinterae*** Boed. Mexico, Nuevo León, *S. Arias 1870* (MEXU): OK340280/ OK340343/ OK340404/ OK340457/ OK340515; ***Mammillariazephyranthoides*** Scheidw. Mexico, San Luis Potosí, *T. Terrazas 887* (MEXU): OK340281/ OK340344/ OK340405/ OK340458/ OK340516; ***Mammilloydiacandida*** (Scheidw.) Buxb. Mexico, San Luis Potosí, *T. Terrazas 885* (MEXU): OK340282/ OK340345/ OK340406/ OK340459/ OK340517; ***Neolloydiaconoidea*** (DC.) Britton & Rose. Mexico, Nuevo León, *T. Terrazas 843* (MEXU): MK449048/ MK449103/ MK449293/ MK449168/ MK449231; ***Neolloydiamatehualensis*** Backeb. Mexico, San Luis Potosí, *B. Vázquez 2551* (MEXU): OK340283/ OK340346/ OK340407/ –/ OK340518; ***Obregoniadenegrii*** Frič, México, Tamaulipas, *H. Sánchez-Mejorada 3670* (MEXU): MK449049/ MK449104/ MK449294/ MK449169/ MK449232; ***Ortegocactusmacdougallii*** Alexander. Mexico, Oaxaca, *S. Arias 483* (MEXU): MK449050/ MK449105/ MK449295/ MK449170/ MK449233; ***Pediocactussimpsonii*** (Engelm.) Britton & Rose. cult. s.n. (JB Instituto de Biología, UNAM), ND: MK449019/ MK449106/ MK449296/ MK449171/ MK449234; ***Pelecyphoraaselliformis*** Ehrenb. Mexico, San Luis Potosí, *H. Sánchez-Mejorada 3610* (MEXU): MK449051/ MK449107/ MK449297/ MK449172/ MK449235; ***Pelecyphorastrobiliformis*** (Werderm.) Frič & Schelle ex Kreuz. Mexico, Nuevo León, *H. Sánchez-Mejorada 3844* (MEXU): OK340284 / OK340347/ MK284097/ OK340460/ MK284157; ***Rapicactusmandragora*** (Frič ex A.Berger) Buxb. & Oehme. Mexico, Coahuila, *U. Guzmán 1445* (MEXU): MK449052/ MK449126/ MK449316/ MK449190/ MK449252; ***Sclerocactusbrevihamatus*** (Engelm.) D.R.Hunt. DES 1989-0315-0101 (DES), ND: –/ –/ –/ AF267964/–. Mexico, Nuevo León, *T. Hernández 68* (MEXU): HM041770/ –/ –/ –/ HM041351; ***Sclerocactusglaucus*** (K.Schum.) L.D.Benson. Schwabe & al. (2015) (ND), United States Colorado: –/ –/ –/ –/ KJ958760; ***Sclerocactusintertextus*** (Engelm.) N.P.Taylor. cult. DES 1993-0823-1001 (DES), ND: HM041683/ –/ –/ HM041417/ HM041263; ***Sclerocactusmariposensis*** (Hester) N.P. Taylor. Mexico, Coahuila, *D. Aquino* 343 (MEXU): OK340268/ OK340331/ MK284098/ OK340445/ MK284158; ***Sclerocactusparviflorus*** Clover & Jotter. Schwabe & al. (2015) (ND), United States Colorado: –/ –/ –/ –/ KJ958785; ***Sclerocactusscheeri*** (Salm-Dyck) N.P.Taylor. Mexico, Coahuila, *T. Terrazas 903* (MEXU): MK449053/ MK449108/ MK449298/ MK449173/ MK449236; ***Sclerocactusspinosior*** (Engelm.) D.Woodruff & L.D.Benson. *Hughes 2* (ISC), ND: –/ –/ –/ AF267965/ –; ***Sclerocactusunguispinus*** (Engelm.) N.P.Taylor. Mexico, Durango, *S. Arias 1902* (MEXU): OK340269/ OK340332/ OK340393/ OK340446/ OK340504; ***Sclerocactuswarnockii*** (L.D.Benson) N.P.Taylor. Mexico, Chihuahua, *S. Arias 2089* (MEXU): OK340270/ OK340333/ OK340394/ OK340447/ OK340505; ***Sclerocactuswhipplei*** (Engelm. & J.M.Bigelow) Britton & Rose. DES 1993-0925-0103 (DES), ND: –/ –/ –/ AF267966/ –; ***Thelocactusbicolor*** (Galeotti ex Pfeiff.) Britton & Rose. Mexico, Coahuila, *T. Terrazas 895* (MEXU): OK340285/ OK340348/ OK340408/ OK340461/ OK340519.

## ﻿Appendix 2

**Table A1.** Insertion-deletion events coded in the alignment for each sequence. Deletion=DEL, insertion=INS, simple sequence repetition (SSR).

Sequence	Event	Sites	Sequence	Event	Sites
*matk*	INS	675-677	*rpl16*	DEL	939-957
*psbA-trnH*	DEL	96-109	*rpl16*	INS	1079-1082
*psbA-trnH*	Del	110-154	*rpl16*	SSR	1148-1150
*psbA-trnH*	Del	127-138	*trnL-F*	INS	365-385
*psbA-trnH*	Del	132-138	*trnL-F*	DEL	390-609
*psbA-trnH*	Del	170-179	*trnL-F*	DEL	345-592
*psbA-trnH*	INS	383-389	*trnL-F*	INS	438-442
*psbA-trnH*	SSR	214-217	*trnL-F*	DEL	453-521
*psbA-trnH*	INS	222	*trnL-F*	SSR	483-484
*psbA-trnH*	DEL	272-364	*trnL-F*	SSR	540-553
*psbA-trnH*	INS	343	*trnL-F*	DEL	848-855
*psbA-trnH*	DEL	362-371	*trnL-F*	Del	853-890
*rpl16*	DEL	30-44	*trnL-F*	DEL	871-1117
*rpl16*	DEL	210-213	*trnL-F*	INS	894-897
*rpl16*	DEL	278-280	*trnL-F*	DEL	1049-1057
*rpl16*	INS	550-567	*trnL-F*	SSR	1151-1152
*rpl16*	SSR	733-738	*trnL-F*	SSR	1205-1209
